# Patch clamp-assisted single neuron lipidomics

**DOI:** 10.1038/s41598-017-05607-3

**Published:** 2017-07-13

**Authors:** Collin B. Merrill, Abdul Basit, Andrea Armirotti, Yousheng Jia, Christine M. Gall, Gary Lynch, Daniele Piomelli

**Affiliations:** 1 Department of Anatomy and Neurobiology, University of California, Irvine, Irvine, CA 92697 USA; 2 Department of Drug Discovery and Development, Istituto Italiano di Tecnologia, Genoa, 16163 Italy; 3 Department of Neurobiology and Behavior, University of California, Irvine, Irvine, CA 92697 USA; 4 Department of Psychiatry and Human Behavior, University of California, Irvine, Irvine, CA 92697 USA; 5 Department of Pharmacology, University of California, Irvine, Irvine, CA 92697 USA; 6 Department of Biological Chemistry, University of California, Irvine, Irvine, CA 92697 USA

## Abstract

Our understanding of the physiological and pathological functions of brain lipids is limited by the inability to analyze these molecules at cellular resolution. Here, we present a method that enables the detection of lipids in identified single neurons from live mammalian brains. Neuronal cell bodies are captured from perfused mouse brain slices by patch clamping, and lipids are analyzed using an optimized nanoflow liquid chromatography/mass spectrometry protocol. In a first application of the method, we identified more than 40 lipid species from dentate gyrus granule cells and CA1 pyramidal neurons of the hippocampus. This survey revealed substantial lipid profile differences between neurons and whole brain tissue, as well as between resting and physiologically stimulated neurons. The results suggest that patch clamp-assisted single neuron lipidomics could be broadly applied to investigate neuronal lipid homeostasis in healthy and diseased brains.

## Introduction

Mammalian brains contain millions to billions of neurons, subdivided into many morphologically and functionally distinct classes^1^. To decode this diversity, neuroscience has deployed an array of strategies that range from the neuron-staining reaction introduced by Camillo Golgi in 1873^2^ to the molecular techniques currently used to profile gene transcription and manipulate activity of specific neuronal subpopulations^3–5^. Single-cell transcriptomics, in particular, has opened an unprecedented window on neuronal diversity by allowing patterns of gene expression in individual neurons to be correlated with structure and function^6^. One key appeal of this approach is that it combines two techniques – patch-clamp and reverse transcriptase-polymerase chain reaction (RT-PCR) – that are commonly used in neuroscience and can therefore be implemented with relative ease.

Lipids intervene, as both structural and signaling molecules, in a wide spectrum of neuronal processes, including ion-channel regulation, vesicle secretion and synaptic plasticity^7–9^. Moreover, disruptions in lipid homeostasis contribute in important ways to neurodegenerative disorders such as Alzheimer’s disease^10^ and Parkinson’s disease^11^, and to neuropsychiatric disorders such as schizophrenia and depression^12^. Present knowledge about neural lipids almost exclusively derives from studies on gross anatomical areas of the brain, in which heterogeneous neuronal populations are intermingled with approximately equal numbers of glial and endothelial cells^1^, or on neuronal and glial cells in primary cultures, which are developmentally immature and are maintained in artificial media that can strongly influence their lipid composition. To address the need for greater cellular specificity, researchers have turned to imaging mass spectrometry (IMS), which permits the simultaneous identification and localization of a broad range of molecules, including lipids, on the surface of biological samples^13^. This approach has provided important insights on the spatial organization of lipid species in giant neurons of the marine mollusk, *Aplysia californica* ^14^, and multicellular preparations of mammalian neural tissue^15, 16^. Nevertheless, IMS suffers from limitations that include, most critically, limited analyte coverage and instrument response linearity, as well as lack of cellular resolution and front-end analyte separation^13^. The present report describes an approach in which lipid profiling of individual neurons is achieved by combining patch-clamp with an optimized nanoflow liquid chromatography/high-resolution mass spectrometry (nLC/MS) protocol. Application of the new method reveals significant lipidome-wide differences between individual neurons of the hippocampus and their surrounding parenchyma, as well as between resting and stimulated neurons.

## Results and Discussion

**Figure 1:**
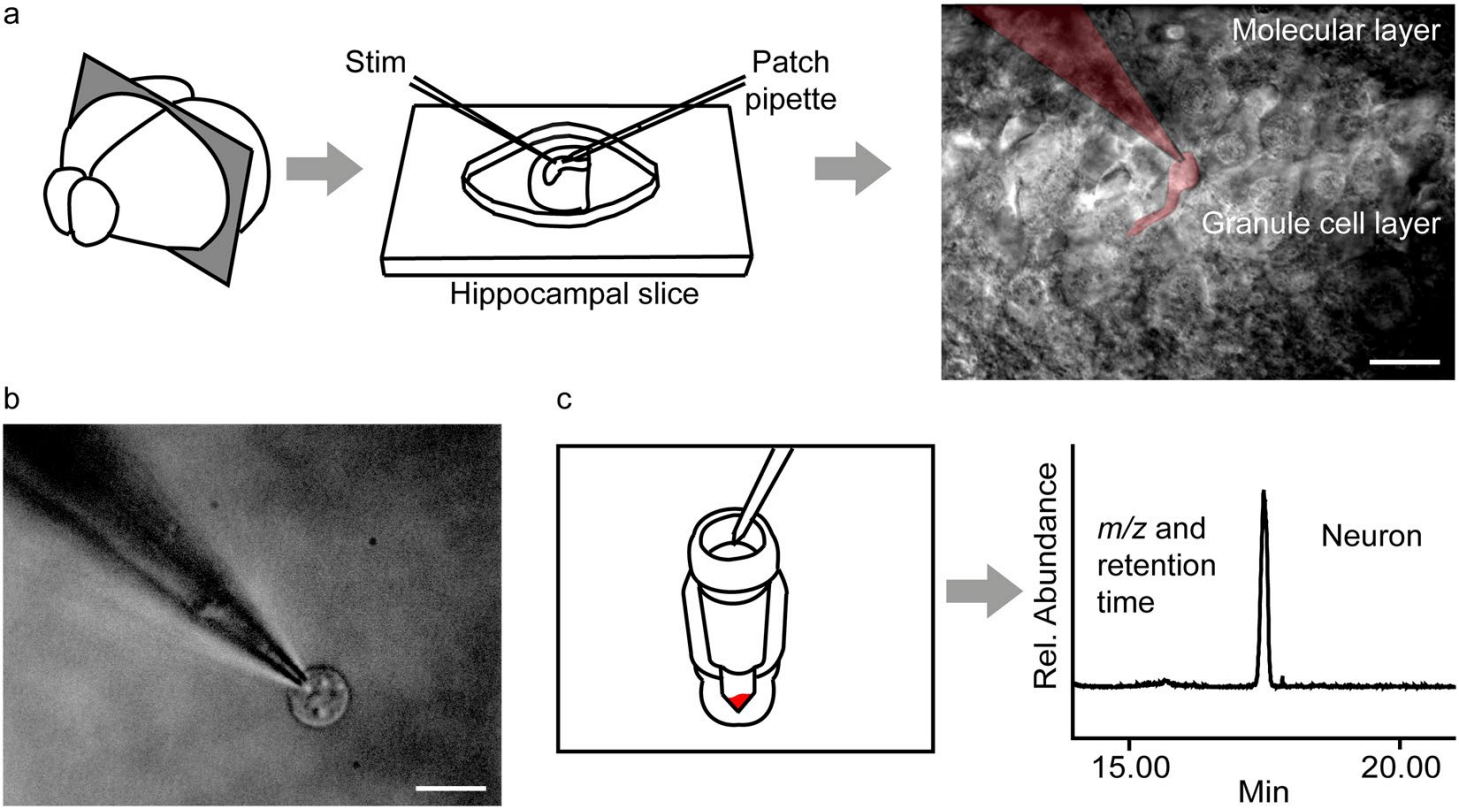
Patch clamp-assisted single neuron lipidomics. (**a**) Mouse brain slices are prepared using a vibratome and transferred to the perfusion chamber of a standard electrophysiology setup. A target neuron is visually identified and patched in the cell-attached configuration using a standard manipulator. A patched granule cell of the dentate gyrus and the attached pipette are shown in pseudocolor. (**b**) The neuronal soma is carefully pulled away from the slice while maintaining the giga-ohm seal. Visual inspection is used to confirm that the neuron is free of extraneous contaminating material such as glial cells; calibration bar: 10 μm (**c**) The patched neuron is aspirated into the pipette tip and transferred to a sterile vial kept in dry ice. Stimulated cells are collected from the slice within 3 min of stimulation. Samples are subjected to single-phase isopropanol extraction and lipids are analyzed by nanoflow liquid chromatography/high-resolution time-of-flight mass spectrometry (nLC-MS).

We used a technique originally devised for single-cell transcriptomics to capture live neurons from acutely dissected mouse hippocampal slices. The slices were prepared with a vibratome and perfused with artificial cerebrospinal fluid (ACSF) in a submerged chamber **(**Fig. 1a). Neurons were identified by shape and location, and patched with the aid of a micromanipulator without rupturing the cell membrane (cell-attached patch mode). To demonstrate the method we selected two classes of neurons – dentate gyrus (DG) granule cells and CA1 pyramidal cells – because they *(i)* differ from one another in size, morphology and biophysical properties^17^; *(ii)* undergo profound structural and functional changes in response to increased excitatory afferent activity^18, 19^; and *(iii)* are part of a reentrant neural circuit that underpins long-term memory formation^20, 21^. Using the micromanipulator, the patched neurons were gently pulled away from the slices while keeping the giga-ohm seal intact and were visualized to ensure that a single cell had been captured and no glial contamination was present (Fig. 1b). The neurons were aspirated into the pipette tip, and expelled into a glass vial kept on dry ice. Samples were subjected to single-phase isopropanol extraction and analyzed by nLC/MS using on-column injection (Fig. 1c). Special precautions were taken to overcome problems generated by the presence of inorganic salts, as described in the Materials and Methods section.

**Figure 2:**
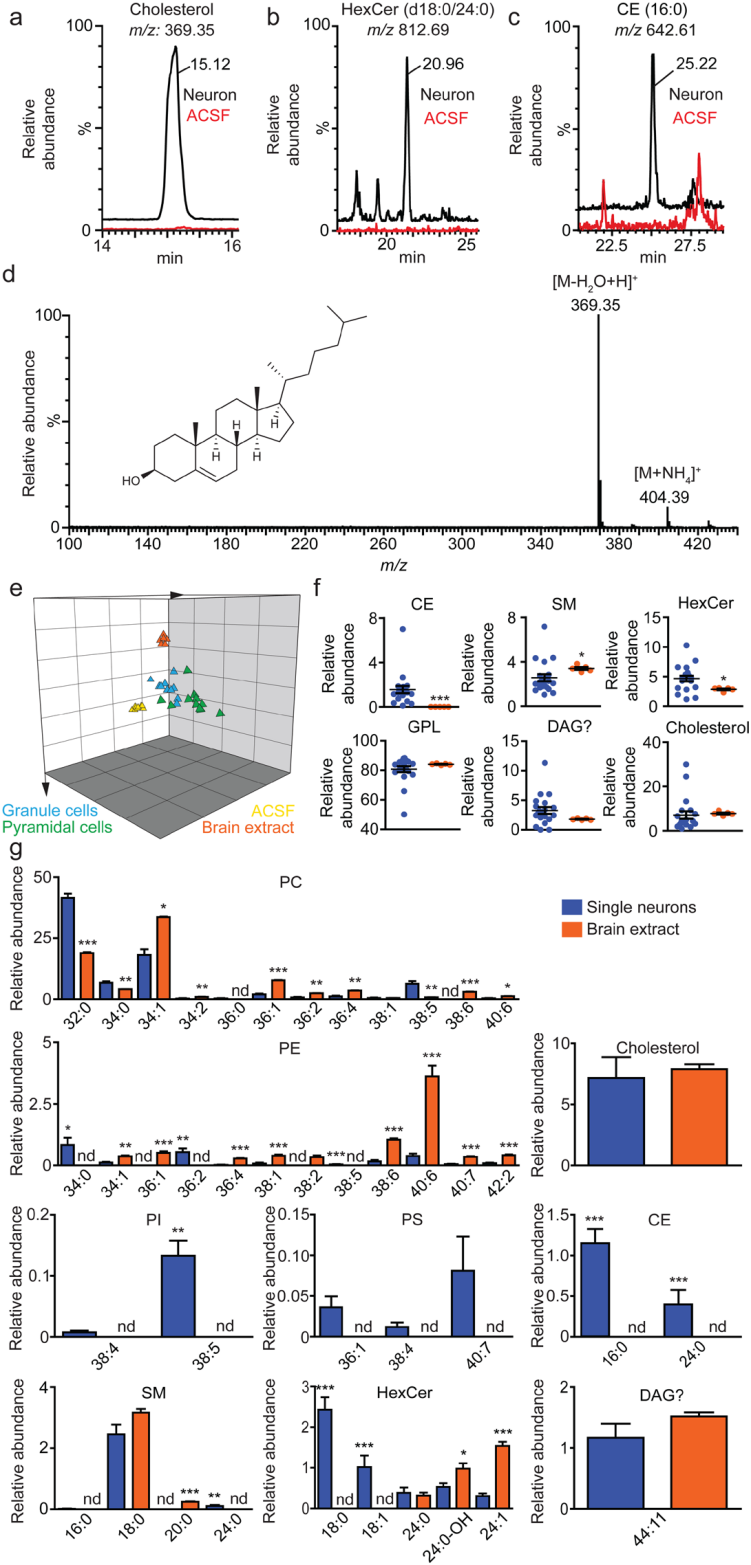
Lipidomics analysis of individual neurons from mouse hippocampus. Representative nLC/MS tracings for (**a**) cholesterol ([M−H_2_O + H]^+^, *m/z* = 369.35, Rt = 15.12 min), (**b**) hexosylceramide (d18:1/24:0) ([M + H]^+^, *m/z* = 812.69, Rt = 20.96), and (**c**) cholesterol ester 16:0 ([M + NH_4_]^+^, *m/z* = 642.61, Rt = 25.22) obtained from a single DG granule cell. Black tracings: neurons; red tracings: artificial cerebrospinal fluid (ACSF). (**d**) Mass spectrum of cholesterol from a single DG granule cell. (**e**) Principal component analysis of lipids present in whole hippocampal tissue (orange triangles), single DG granule cells (blue triangles), single CA1 pyramidal cells (green triangles) and ACSF (yellow triangles). (**f**) Relative quantification of main lipid classes from individual neurons (blue circles; granule and pyramidal cells combined) and whole hippocampal tissue (orange circles). Abbreviations: CE, cholesteryl esters; DAG?, a lipid tentatively identified as diacylglycerol; GPL, glycerophospholipids; HexCer, hexosylceramides; SM, sphingomyelins. (**g**) Relative quantification of lipid species from individual neurons (blue bars) and whole hippocampal tissue (orange bars). Results are represented as mean ± s.e.m (n = 20 single neurons and 5 punches from different slices per group); *P < 0.05; **P < 0.005; ***P < 0.001; Mann-Whitney U test.

Total ion current (TIC) was monitored in the positive-ion mode and spectra were acquired in the *m/z* range = 100–1000. TIC tracings of single-neuron extracts were uninformative. Nevertheless, considering that relevant signals could be hidden by matrix-derived noise, we extracted ions for a representative set of 236 lipids known to be present in neural tissue **(**Supplementary Table S1 **)**. We focused on lipid-related signals that met the following criteria: (*i*) signal-to-noise ratio >3; (*ii*) presence in ≥75% of single-neuron samples; (*iii*) peak area at least 3x higher than two sets of controls including freshly prepared ACSF (5 μL) and ‘sham-capture’ controls, in which a patch pipette was lowered into the slice but no cell was collected. Table 1 lists 41 analytes that met the filtering criteria. These included abundant membrane constituents such as cholesterol (Fig. 2a), but also less-represented species such as hexosylceramide (d18:1/24:0) and cholesteryl ester (CE) 16:0 (total number of carbons: total number of double bonds) (Fig. 2b,c). For a few neuronal lipids we were able to obtain full mass spectra (e.g., cholesterol, Fig. 2d **)**. In most cases, however, identification relied on the comparison with retention time (Rt) and accurate mass/charge (*m/z*) values (with 5 ppm accuracy) of lipids represented in highly diluted extracts (1:50,000) of whole mouse hippocampus analyzed under identical nLC/MS conditions (Supplementary Figs.  S1–40).

**Table 1:** Lipid species detected in individual hippocampal neurons.

	Lipid species	Formula	Adduct Type	Calculated *m/z*	Observed *m/z*	Mass Error (ppm)	RT (min)
Glycerophospholipids	PC (32:0)	C_40_H_80_NO_8_P	[M + H]^+^	734.5694	734.5693	0.0	16.7
	PC (34:0)	C_42_H_84_NO_8_P	[M + H]^+^	762.6007	762.6003	−0.5	18.3
	PC (34:1)	C_42_H_82_NO_8_P	[M + H]^+^	760.5851	760.5847	−0.5	16.9
	PC (34:2)	C_42_H_80_NO_8_P	[M + H]^+^	758.5694	758.5675	−2.5	15.7
	PC (36:0)	C_44_H_88_NO_8_P	[M + H]^+^	790.6320	790.6294	−3.3	19.8
	PC (36:1)	C_44_H_86_NO_8_P	[M + H]^+^	788.6164	788.6151	−1.6	18.4
	PC (36:2)	C_44_H_84_NO_8_P	[M + H]^+^	786.6007	786.5988	−2.4	17.0
	PC (36:4)	C_44_H_80_NO_8_P	[M + H]^+^	782.5694	782.5728	4.3	15.4
	PC (38:1)	C_46_H_90_NO_8_P	[M + H]^+^	816.6477	816.6494	2.1	19.8
	PC (38:5)	C_46_H_82_NO_8_P	[M + H]^+^	808.5851	808.5848	−0.4	15.6
	PC (38:6)	C_46_H_80_NO_8_P	[M + H]^+^	806.5694	806.5730	4.5	15.0
	PC (40:6)	C_48_H_84_NO_8_P	[M + H]^+^	834.6007	834.5994	−1.6	16.4
	PE (34:0)	C_39_H_78_NO_8_P	[M + H]^+^	720.5538	720.5514	−3.3	19.4
	PE (34:1)	C_39_H_76_NO_8_P	[M + H]^+^	718.5381	718.5367	−1.9	16.9
	PE (36:2)	C_41_H_78_NO_8_P	[M + H]^+^	744.5538	744.5510	−3.8	17.1
	PE (36:4)	C_41_H_74_NO_8_P	[M + H]^+^	740.5225	740.5236	1.5	15.6
	PE (38:1)	C_43_H_84_NO_8_P	[M + H]^+^	774.6007	774.6045	4.9	17.6
	PE (38:5)	C_43_H_76_NO_8_P	[M + H]^+^	766.5381	766.5377	−0.5	15.7
	PE (38:6)	C_43_H_74_NO_8_P	[M + H]^+^	764.5225	764.5261	4.7	15.2
	PE (40:1)	C_45_H_88_NO_8_P	[M + H]^+^	802.6320	802.6302	−2.2	19.1
	PE (40:6)	C_45_H_78_NO_8_P	[M + H]^+^	792.5538	792.5529	−1.1	16.7
	PE (40:7)	C_45_H_76_NO_8_P	[M + H]^+^	790.5381	790.5403	2.8	15.4
	PE (42:2)	C_47_H_90_NO_8_P	[M + H]^+^	828.6477	828.6461	−1.9	18.9
	PI (38:4)	C_47_H_83_O_13_P	[M + NH_4_]^+^	904.5910	904.5955	5.0	15.0
	PI (38:5)	C_47_H_81_O_13_P	[M + NH_4_]^+^	902.5753	902.5710	−4.8	13.8
	PS (36:1)	C_42_H_80_NO_10_P	[M + H]^+^	790.5593	790.5565	−3.5	15.2
	PS (38:4)	C_44_H_78_NO_10_P	[M + H]^+^	812.5436	812.5456	2.2	16.1
	PS (40:7)	C_46_H_76_NO_10_P	[M + H]^+^	834.5279	834.5239	−4.8	18.0
Sphingolipids	HexCer (d18:1/18:0)	C_42_H_81_NO_8_	[M + H]^+^	728.6034	728.6070	5.0	16.8
	HexCer (d18:1/18:1)	C_42_H_81_NO_8_	[M + H]^+^	726.5878	726.5850	−3.9	16.4
	HexCer (d18:1/24:0)	C_48_H_93_NO_8_	[M + H]^+^	812.6973	812.6939	−4.2	21.0
	HexCer (d18:1/24:0-OH)	C_48_H_93_NO_9_	[M + H]^+^	828.6923	828.6912	−1.3	20.6
	HexCer (d18:1/24:1)	C_48_H_93_NO_8_	[M + H]^+^	810.6817	810.6820	0.4	19.6
	SM (d18:1/16:0)	C_39_H_79_N_2_O_6_P	[M + H]^+^	703.5748	703.5760	1.7	15.2
	SM (d18:1/18:0)	C_41_H_83_N_2_O_6_P	[M + H]^+^	731.6061	731.6082	2.9	18.7
	SM (d18:1/24:0)	C_47_H_95_N_2_O_6_P	[M + H]^+^	815.7000	815.6962	−4.6	21.7
Sterol lipids	CE (16:0)	C_43_H_76_O_2_	[M + NH_4_]^+^	642.6183	642.6160	−3.6	25.2
	CE (24:0)	C_51_H_92_O_2_	[M + NH_4_]^+^	754.7446	754.7464	2.4	27.6
	Cholesterol	C_27_H_46_O	[M − H_2_O + H]^+^	369.3515	369.3531	4.3	15.1
Glycerolipids	DG (42:10)	C_45_H_68_O_5_	[M + NH_4_]^+^	706.5405	706.5416	1.3	15.1
	DG (44:11)	C_47_H_70_O_5_	[M + NH_4_]^+^	732.5561	732.5585	3.3	15.3

We normalized the abundance of individual lipid species by the total abundance of all identified lipids and used principal component analysis to compare neurons to whole hippocampal tissue, as well as DG granule cells to CA1 pyramidal cells. The analyses did not reveal significant differences between neuron types (Supplementary Fig. S41), but clearly separated neurons (granule and pyramidal cells, separated or combined) from tissue (Fig. 2e). The two latter datasets differed in three main lipid classes – CE (*P* = 0.0007), sphingomyelins (SM) (*P* = 0.0451) and hexosylceramides (HexCer) (*P* = 0.0383) (Fig. 2f) – but also in specific members of all classes (Fig. 2g). These included, among others, phosphatidylcholine (PC) 32:0 (*P* = 0.0008), PC 34:1 (*P* = 0.0273); phosphatidylethanolamine (PE) 38:6 (*P* = 0.0006), PE 40:6 (*P* = 0.0001); CE 16:0 (*P* = 0.0007), CE 24:0 (*P* = 0.0007), phosphatidylinositol (PI) 38:5 (*P* = 0.0013), SM 20:0 (*P* = 0.0001), SM 24:0 (*P* = 0.0001), HexCer (d18:1/24:1) (*P* = 0.0013) and a lipid tentatively identified as diacylglycerol (DAG?) 42:10 (*P* = 0.0446; Supplementary Fig. S39). Most notably, neurons contained substantially higher levels of CE 16:0, CE 24:0, PI 38:5 and SM 24:0 than did whole hippocampal tissue (Fig. 2g). While the biological significance of these differences is presently unclear, the finding that the lipid profile of adult brain neurons is distinguishable from that of brain parenchyma emphasizes the need to investigate lipid homeostasis at cellular resolution.

Stimulation of excitatory afferent fibers causes long-lasting morphological and functional changes in hippocampal neurons^17, 20–22^. To test whether neural activity also influences the cells’ lipid profile, we placed electrodes either in the lateral perforant path (LPP) or the Schaffer-commissural (SC) systems, projections that provide excitatory input to DG granule cells and CA1 pyramidal cells, respectively^17, 20, 21^. In both cases, we used a stimulation pattern that mimicked high-frequency gamma waves, which are generated during cognitive processing^23^ and elicit long-term potentiation (LTP) of synaptic transmission^21, 22, 24^. Neuronal cell bodies were collected within 3 min of stimulation, snap-frozen and subjected to analysis. In parallel experiments, to assess the impact of stimulation on whole DG tissue, we used a manual puncher to remove cylinders (0.86 mm diameter) from the DG of control and stimulated slices.

**Figure 3:**
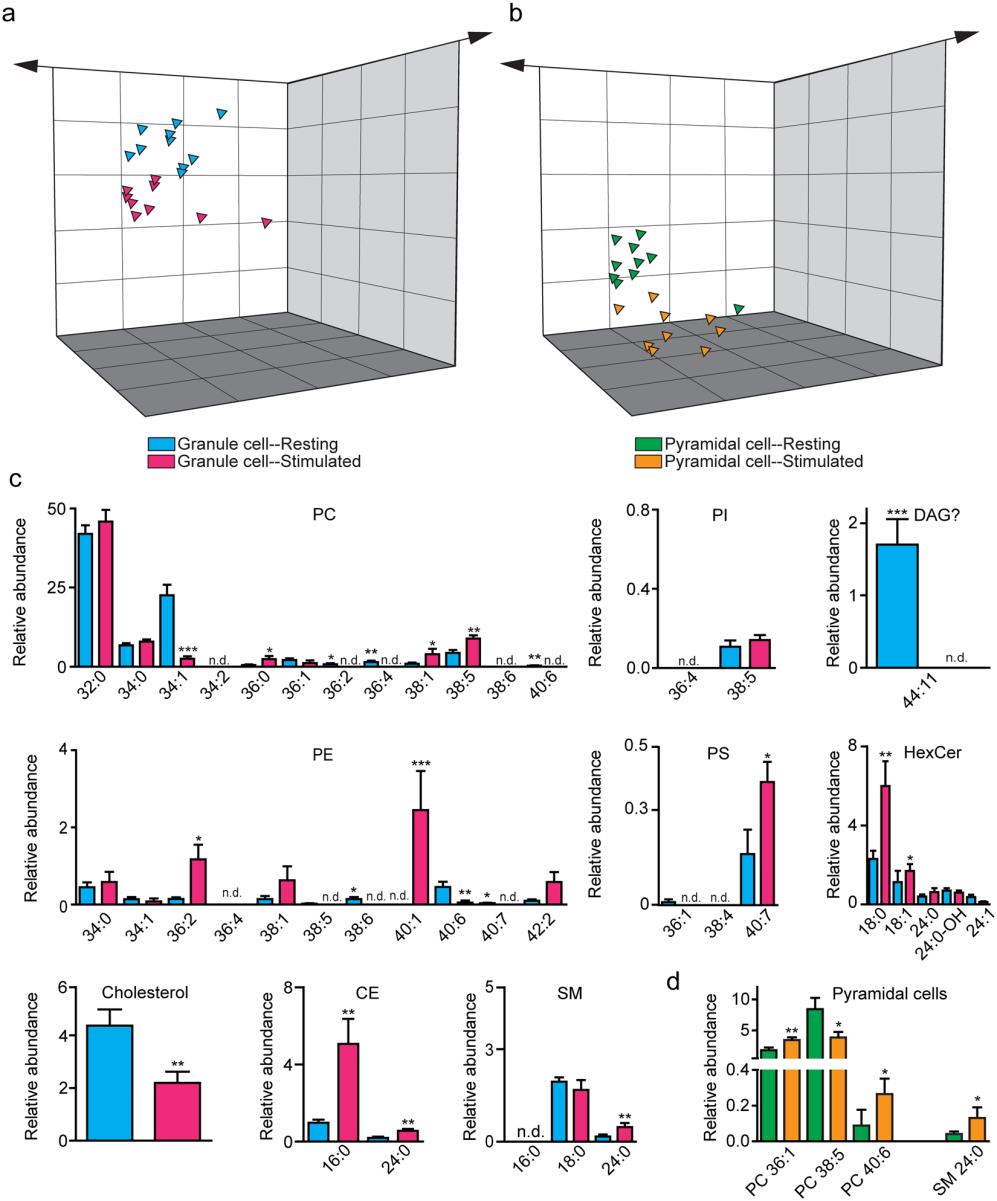
Effects of physiological stimulation on the lipidome of individual hippocampal neurons. Principal component analysis of lipids from resting and stimulated **(a)** DG granule cells (blue: resting; magenta: stimulated) or **(b)** CA1 pyramidal cells (green: resting; orange: stimulated). Relative quantification of individual lipid species from **(c)** resting (blue bars) or stimulated (magenta bars) granule cells; and **(d)** resting (green bars) or stimulated (orange bars) pyramidal cells. Results are represented as mean ± s.e.m (n = 10 single neurons per group); *P < 0.05; **P < 0.005; ***P < 0.001; Mann-Whitney U test.

LPP stimulation substantially altered the lipid profile of DG granule cells (Fig. 3a). The changes were bidirectional and involved multiple lipid species. As shown in Fig. 3c, significant increases were seen with PC 36:0 (*P* = 0.0127), PC 38:1 (*P* = 0.0133), PC 38:5 (*P* = 0.0042), PE 36:2 (*P* = 0.0115), PE 40:1 (*P* = 0.0001), phosphatidylserine (PS) 40:7 (*P* = 0.0452), HexCer (d18:1/18:0) (*P* = 0.0098), HexCer (d18:1/18:1) (*P* = 0.0172), CE 16:0 (*P* = 0.0042), CE 24:0 (*P* = 0.0076), and SM 24:0 (*P* = 0.0076). Decreases were observed instead with PC 34:1 (*P* = 0.0004), tentatively identified DAG 42:10 (*P* = 0.0001) and DAG 44:11 (*P* = 0.0004), PE 40:6 (*P* = 0.0071) and non-esterified cholesterol (*P* = 0.0041). No significant changes were seen in other detectable lipids. Compared with granule cells, enhanced afferent activity had more restricted effects on pyramidal neurons (Fig. 3b), in which levels of PC 36:1 (*P* = 0.0031), PC 40:6 (*P* = 0.0380) and SM 24:0 (*P* = 0.0172) were elevated following stimulation, whereas those of PC 38:5 were lowered (*P* = 0.0457) (Fig. 3d). In sharp contrast with single granule cell neurons, and underscoring the cellular specificity of the response, we found that the lipid profile of DG punches was not significantly affected by LPP stimulation (Supplementary Fig. S42). Collectively, the results of these experiments suggest that physiologically relevant patterns of neural activity cause marked lipidome-wide alterations in individual hippocampal neurons, which are measurable by the present method. Many of these modifications may be attributed to accelerated phospholipid remodeling and cholesterol esterification, and might reflect therefore an activity-dependent reorganization of the neuronal membrane. This conclusion is consistent with evidence indicating that high-frequency afferent activity promotes cholesterol redistribution^25^ and initiates postsynaptic spine enlargement and new spine generation in hippocampal neurons^26, 27^. Similarly, accumulation of SM 24:0 might reflect an expansion of SM-rich liquid-ordered domains (‘lipid rafts’)^28^ – possibly linked to accelerated trafficking of ionotropic glutamate receptors^29–31^ – and increased coupling between inner and outer membrane leaflets^32^. Testing these hypotheses will require, of course, dedicated experiments.

In sum, the patch clamp-assisted lipidomics method described here allowed us to detect lipids in individual neurons freshly isolated from live mammalian brains. Because of its sensitivity, flexibility and ease of implementation, the method may be used, alone or in combination with transgenic expression of fluorescent lineage-specific proteins, to profile the lipidome of most, if not all, neurons in the central nervous system. Furthermore, the method can be easily adapted for use in targeted lipidomics, with potential gains in sensitivity and ability to quantify, or combined with RT-PCR to link transcriptomics and lipidomics data at the single neuron level. Its main current weakness – a still limited coverage of the neuronal lipidome – should be progressively mitigated by improvements in LC-MS/MS technology. Thus, the method is applicable to a broad variety of studies of lipid homeostasis in the healthy and diseased brain.

## Materials and Methods

### Reagents, standards and instruments

Solvents and chemicals were purchased from Sigma Aldrich. Instruments, columns and data analysis software were from Waters Inc., unless otherwise indicated.

### Slice preparation

All experimental protocols were approved by the University of California Irvine Institutional Animal Care and Use Committee and were carried out in accordance with NIH guidelines for the use of experimental animals. Male CD1 mice (16–28 days old) were used. Animals were deeply anesthetized with isoflurane and decapitated. The brains were quickly removed and sectioned into 400 μm-thick slices. Horizontal sections were used for experiments with dentate gyrus (DG) granule cells and coronal sections were used for experiments with CA1 pyramidal cells. The slices were placed on a submerged net and incubated for 1 h in artificial cerebrospinal fluid (ACSF) containing (in mM) 119 NaCl, 26 NaHCO_3_, 2.5 KCl, 1.0 NaH_2_PO_4_, 2.5 CaCl_2_, 2.7 MgSO_4_, and 11 glucose, saturated with 95% O_2_/5% CO_2_ (pH 7.4). The slices were then transferred to a submerged recording chamber and continuously perfused with ACSF (30 °C) at a flow rate of 2–3 mL/min^22^.

### Electrophysiology

DG granule cells and CA1 pyramidal cells were visually selected using a BX50WI microscope (Olympus) equipped with DIC optics and a digital CCTV camera (Hamamatsu). Cells were patched with a 5–6 MΩ micropipette filled with 2–3 μL of filtered intracellular recording solution containing (in mM) 125 KCl, 2.8 NaCl, 2 MgCl_2_, 10 HEPES, 2 ATP-Mg^2+^, 0.3 GTP-Na, and 100 EGTA-K. Data were recorded with an Axopatch 1-D amplifier (Molecular Devices) and filtered at 5 kHz. Signals were digitized with a Digidata 1440 A digitizer (Molecular Devices) attached to a computer with pClamp 10.3 (Molecular Devices). Slices were stimulated with a bipolar stainless steel electrode (FMC) attached to a Grass photoelectric isolation unit and an S8800 stimulator. The electrodes were positioned either in the lateral perforant path (for dentate gyrus granule cell stimulation) or the Schaffer-commissural system (for CA1 pyramidal cell stimulation). High-frequency stimulation was delivered at 10 μA in two 100 Hz bursts for 100 ms, separated by 20 s^33^.

### Patch clamp-guided neuron capture

Neurons were captured and removed from the slice by application of gentle suction via the patch pipette (without aspirating the cell or disrupting the giga-Ω seal). The pipette and attached cell were gently withdrawn from the slice, keeping the cell attached, until the cell was approximately 200 μm above the slice. The cell was then carefully aspirated into the patch pipette, immediately transferred to a sterile Total Recovery vial (Waters), and snap-frozen on dry ice. Stimulated cells were captured and removed from the slice within 3 min of stimulation. Sham-capture control samples were obtained by dipping a patch pipette (filled with intracellular solution) into the slice and withdrawing it without suction. These samples were also immediately transferred to a sterile vial and snap-frozen in dry ice. All captured samples were stored at −80 °C until analysis.

### Tissue punch collection

Horizontal brain slices containing the hippocampus were prepared as described above and transferred to a submerged recording chamber, where they were continuously perfused with oxygenated ACSF (30 °C) at a flow rate of 2–3 mL/min. The lateral perforant pathway was stimulated using a bipolar stainless steel electrode. High frequency stimulation was applied at 10 μA in 2 bursts at 100 Hz for 100 ms, separated by 20 s. Control slices underwent the same treatment without high-frequency stimulation. A cylinder of DG tissue was collected using a 0.86 mm diameter manual puncher. A second punch was taken from the hippocampus proper for protein quantification. Tissue samples were transferred to glass vials and snap-frozen in dry ice. Lipids were extracted, separated, and analyzed in the same manner as single-neuron samples, as described below. Protein quantification was performed using a Pierce BCA kit (Thermo Fisher), according to the manufacturer protocol. Tissue samples were homogenized in 100 μL of TENT buffer containing 50 mM Tris-HCl (pH 7.0), 1 mM EDTA, 0.15 M NaCl, and 1% Triton-X 100. Absorbance was measured with a SpectraMax plate reader (Molecular Devices) at 562 nm. Each sample was run in triplicate.

### Lipid extraction from single neurons

Most lipid extraction methods only sample a fraction of the lipidome^34^. The following procedure was selected after extensive development because it provided superior recovery and nLC/MS peak shapes when compared to other extraction methods. Neuron and blank samples were subjected to single-phase isopropanol extraction. Using a Hamilton syringe, 5 µl of isopropanol were added to the samples. The samples were mixed for 1 min using a Vortex and 5 µL of mobile phase A (10 mM ammonium formate (pH 5) in 60:40 acetonitrile:water) were added. The samples were then mixed for additional 2 min and centrifuged for 15 min at 3500 rpm at 4 °C. 5 µL of the supernatant were injected in the nano LC-MS system (full loop mode).

### Nano-LC/MS setup

The samples were loaded onto a Nano-Acquity system, equipped with a 150 µm × 150 mm T3 nanobore column. Eluents were: A = 10 mM ammonium formate (pH 5) in 60:40 acetonitrile/water and B = 10 mM ammonium formate (pH 5) in 90:10 isopropanol:water. Flow rate was set to 800 nL/min. The separation was carried out with a linear gradient of solvent B. Initially, 20% B was maintained for 4.5 min and then increased to 60% B in 1 min. % B was then raised from 60% to 100% in the following 25.5 min and maintained at 100% B for additional 5 min. The system was then flushed using a 10 min window at 100% A to help the washout of inorganic salts that may clog the nanospray tip and decrease its lifetime. The column was then re-equilibrated for 5 min at 20% B. Injection volume was set to 5 µl, full loop mode. The system was configured for direct injection mode (no trapping). Eluents were analyzed using a nano-lockspray ion source equipped with an uncoated silica tip (6 cm long, 360 µm external × 20 µm internal diameter) and operating in positive ion mode (ESI+) at 1.8 kV potential. In initial tests, data were also collected in negative-ion mode, but this polarity was subsequently set aside owing to its substantially lower sensitivity. Mass detection was carried out on a G2 qTOF instrument, acquiring spectra in the 100–1000 m/z range in sensitivity mode (V-mode TOF), which enables a 10,000 full-width at half maximum resolution. Cone voltage was set to 40 V. The total MS scan was divided into three experimental sections. In the first section, source cone voltage was set to zero to reduce the entry of inorganic salts into the mass spectrometer; the second section consisted of MS acquisition mode with cone voltage set to 40 V; while in the third section the cone voltage was set back to zero again for column reconditioning. Scan time was 0.3 s. All MS data were recalibrated in real time by acquiring every 30 s a standard leucine enkephaline reference solution (2 ng/ml, continuously infused into the ion source through the lockspray source).

### Data analysis

Lipid species were detected by extracting accurate *m/z* value ion currents (XIC) from the total ion current (TIC) and then repeating the same procedure for a blank control (ACSF, sham capture). Appropriate ion adducts ([M + H]^+^, [M−H_2_O + H]^+^, [M + NH_4_]^+^, [M + Na]^+^) were selected based on published data from lipidmaps.org^35, 36^. Only XIC peaks showing a signal/noise ratio higher than 3 and whose peak areas were at least 3 times higher than the corresponding blank samples were considered. XIC peaks were manually integrated. As MS/MS data could not be acquired on single neuron samples, a reference sample consisting of a highly diluted hippocampal lipid extract (1:50,000) was analyzed along with the single-neuron samples, in order to ensure consistency with the retention times and adduct types of the lipid species observed. The order of acquisition of single neuron samples, system blanks, reference samples and procedure blanks was randomized. Unsupervised data analysis on the 41 positively identified and quantified lipids was performed using the multivariate data analysis tool embedded in MarkerLynx software. For quantitative evaluation, a statistical analysis of the measured peak areas was carried out using a two-tailed Mann-Whitney U test included in Graphpad PRISM 5.03 software (La Jolla, CA, USA). LC-MS data available upon reasonable request.

## Electronic supplementary material

Supplementary Information
