# Author Correction: Patch clamp-assisted single neuron lipidomics

**DOI:** 10.1038/s41598-018-24605-7

**Published:** 2018-04-25

**Authors:** Collin B. Merrill, Abdul Basit, Andrea Armirotti, Yousheng Jia, Christine M. Gall, Gary Lynch, Daniele Piomelli

**Affiliations:** 1 Department of Anatomy and Neurobiology, University of California, Irvine, Irvine, CA 92697 USA; 2 Department of Drug Discovery and Development, Istituto Italiano di Tecnologia, Genoa, 16163 Italy; 3 Department of Neurobiology and Behavior, University of California, Irvine, Irvine, CA 92697 USA; 4 Department of Psychiatry and Human Behavior, University of California, Irvine, Irvine, CA 92697 USA; 5 Department of Pharmacology, University of California, Irvine, Irvine, CA 92697 USA; 6 Department of Biological Chemistry, University of California, Irvine, Irvine, CA 92697 USA

Correction to: *Scientific Reports* 10.1038/s41598-017-05607-3, published online 13 July 2017

The Acknowledgements section in this Article is incomplete.

“This work was funded by a grant from the National Institute on Drug Abuse (to DP) 1R21DA041776-01. We thank Prof. Stephen H. White for thoughtful discussion. We also thank Dr. Robert C. Murphy for verification of lipid identities and technical details of our mass spectrometry methodology.”

should read:

“This work was funded by a grant from the National Institute on Drug Abuse (to DP) 1R21DA041776-01 and an institutional training grant from the National Institute on Neurological Disorders and Stroke 5T32NS045540-14 (which funded CM). We thank Prof. Stephen H. White for thoughtful discussion. We also thank Dr. Robert C. Murphy for verification of lipid identities and technical details of our mass spectrometry methodology.”

