# Supplementary material for: Patch clamp-assisted single neuron lipidomics
Source: Sci Rep. 2017 Jul 13;7:5318. doi: 10.1038/s41598-017-05607-3 (PMC5509708; doi:10.1038/s41598-017-05607-3)
Supplement: Supplementary file 1 — Supplementary Information [file 41598_2017_5607_MOESM1_ESM.pdf]

Patch clamp-assisted single neuron lipidomics

Collin B. Merrill, Abdul Basit, Andrea Armirotti,Yousheng Jia,
Christine M. Gall, Gary Lynch, Daniele Piomelli

SUPPLEMENTARY FIGURES

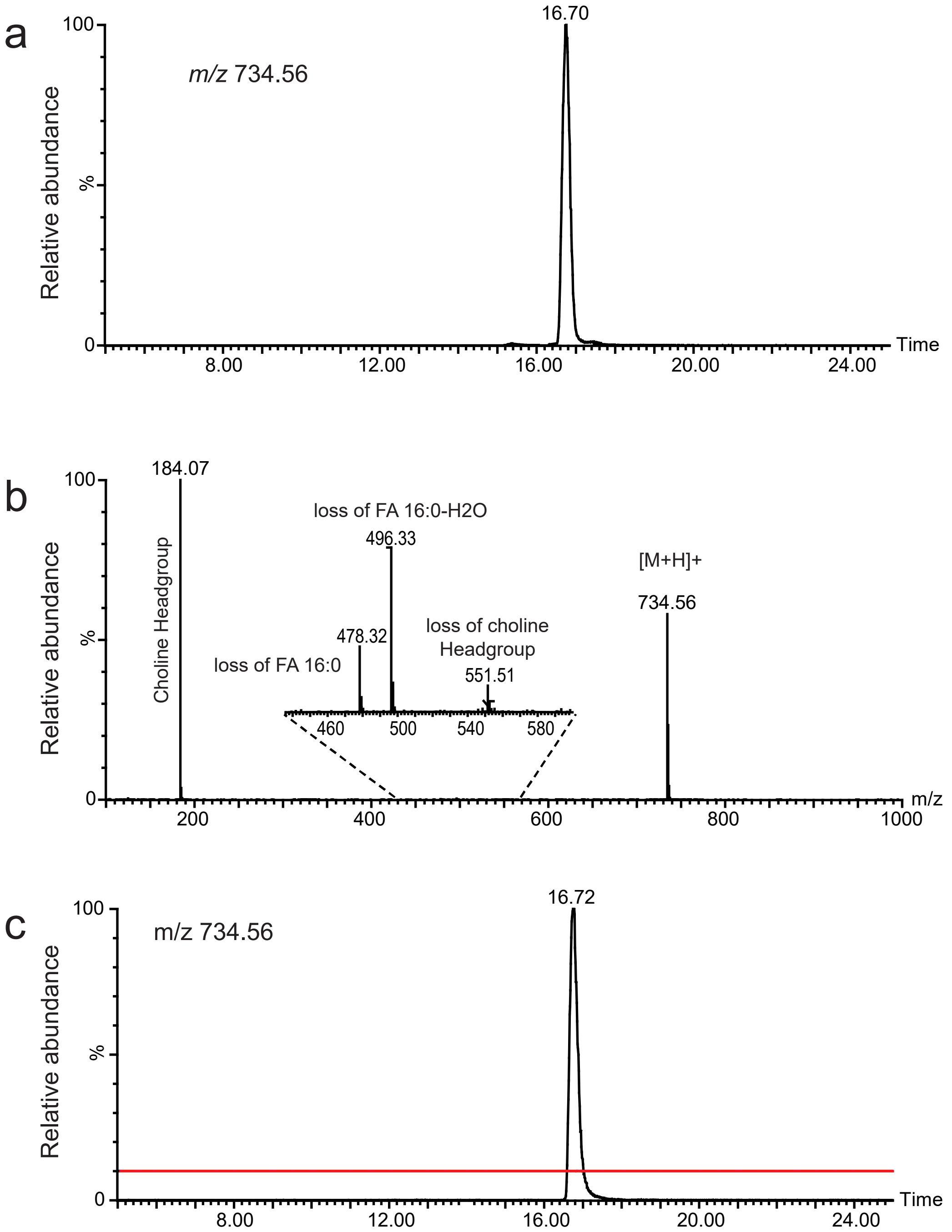

Supplementary Figure S1. Identification of PC 32:0 (*m/z* = 734.56) in diluted extract (1:50,000) of whole hippocampal tissue (A: representative nLC tracing; B: mass spectrum) and isopropanol extract of a single DG granule cell (C: representative nLC tracing). Black tracings: neurons; red tracings: artificial cerebrospinal fluid.

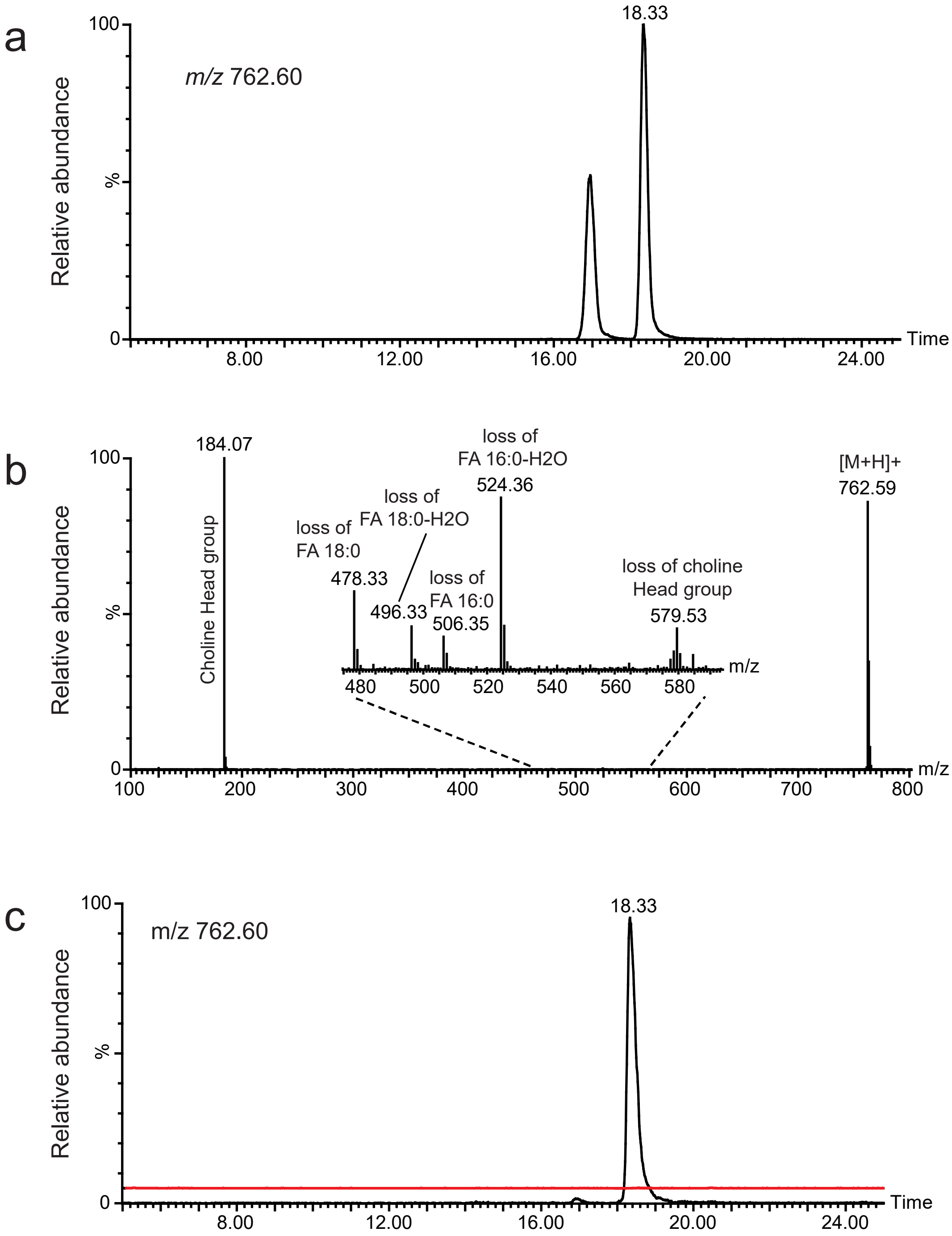

Supplementary Figure S2. Identification of PC 34:0 (*m/z* = 762.60) in diluted extract (1:50,000) of whole hippocampal tissue (A: representative nLC tracing; B: mass spectrum) and isopropanol extract of a single DG granule cell (C: representative nLC tracing). Black tracings: neurons; red tracings: artificial cerebrospinal fluid.

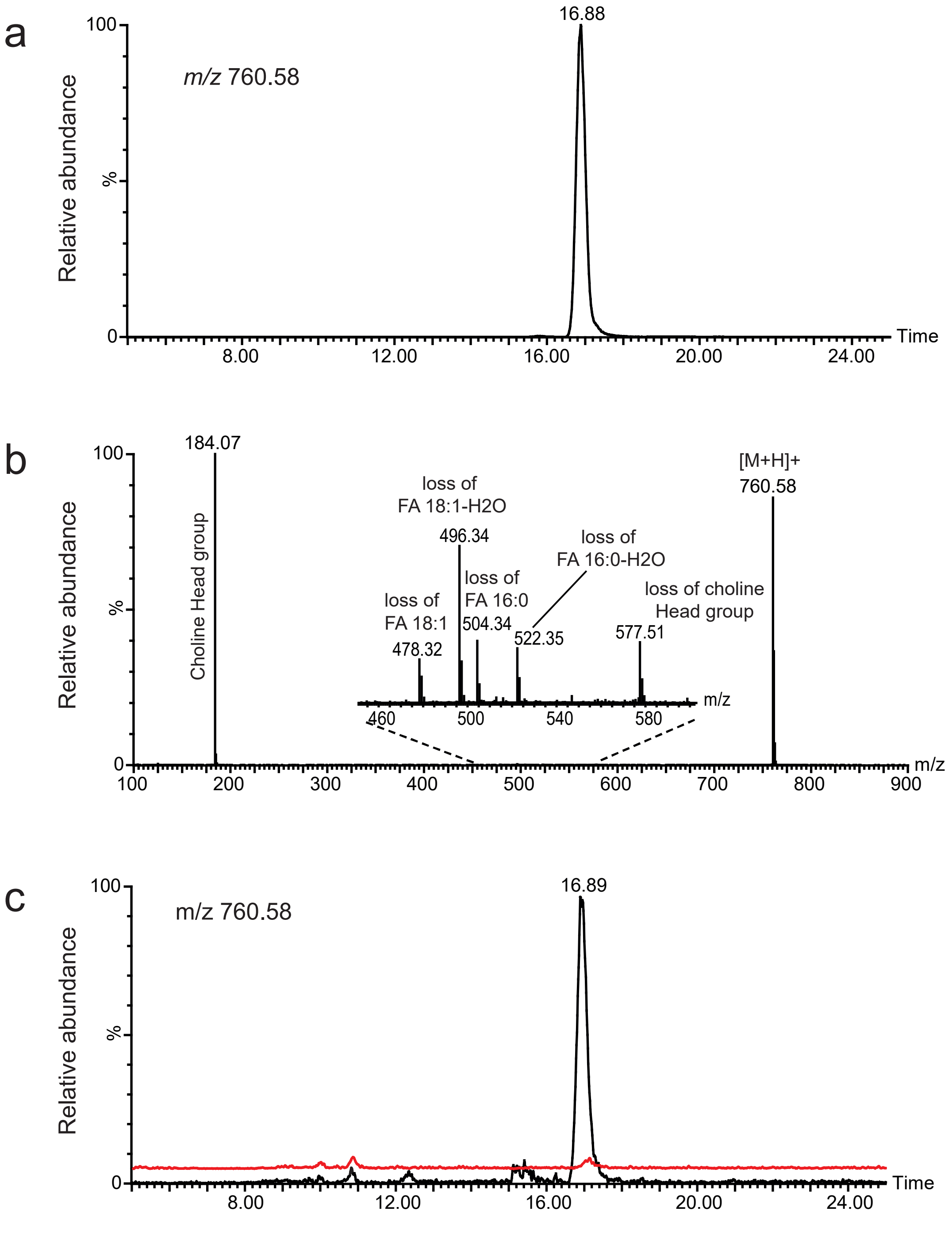

Supplementary Figure S3. Identification of PC 34:1 (*m/z* = 760.58) in diluted extract (1:50,000) of whole hippocampal tissue (A: representative nLC tracing; B: mass spectrum) and isopropanol extract of a single DG granule cell (C: representative nLC tracing). Black tracings: neurons; red tracings: artificial cerebrospinal fluid.

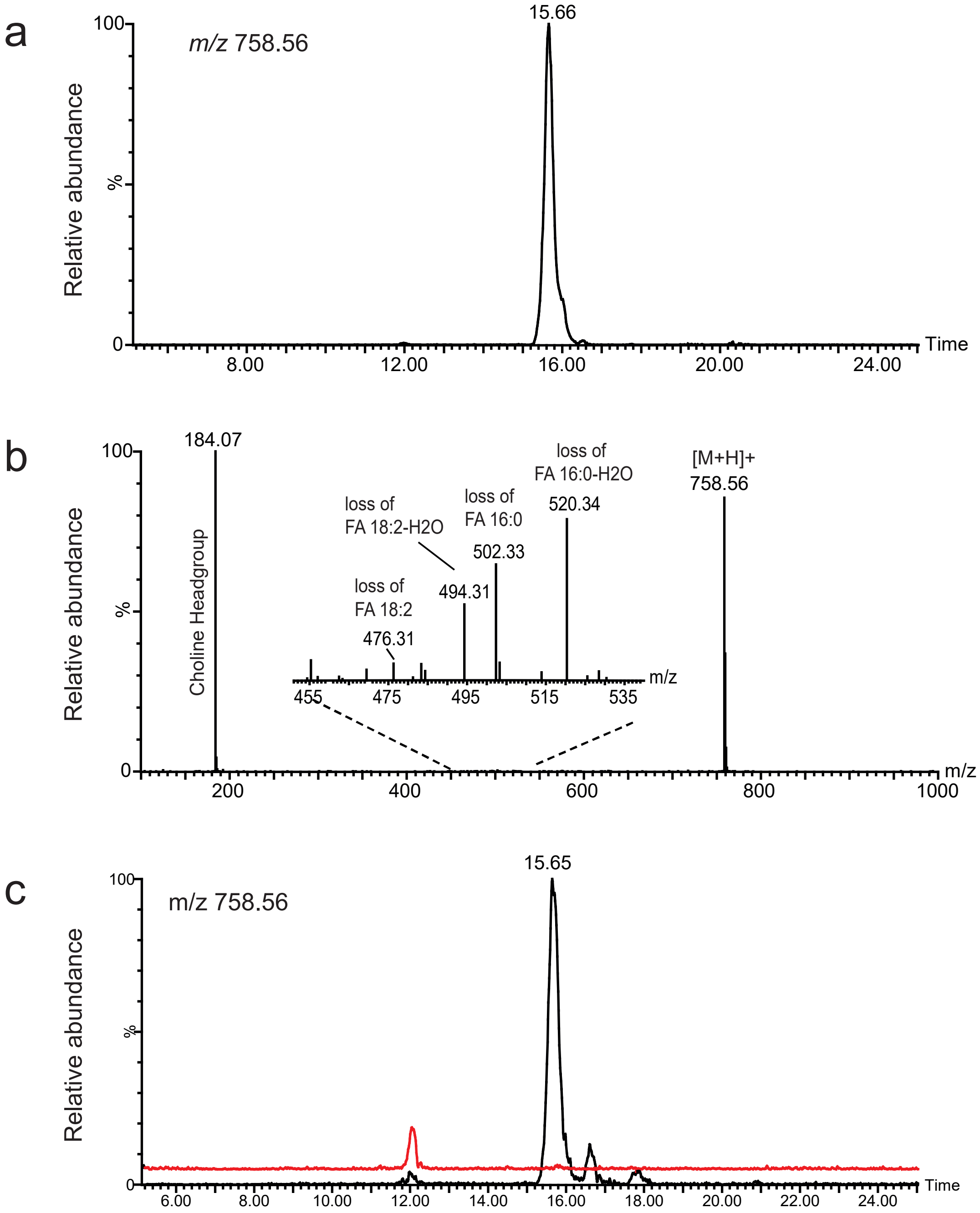

Supplementary Figure S4. Identification of PC 34:2 (*m/z* = 758.56) in diluted extract (1:50,000) of whole hippocampal tissue (A: representative nLC tracing; B: mass spectrum) and isopropanol extract of a single DG granule cell (C: representative nLC tracing). Black tracings: neurons; red tracings: artificial cerebrospinal fluid.

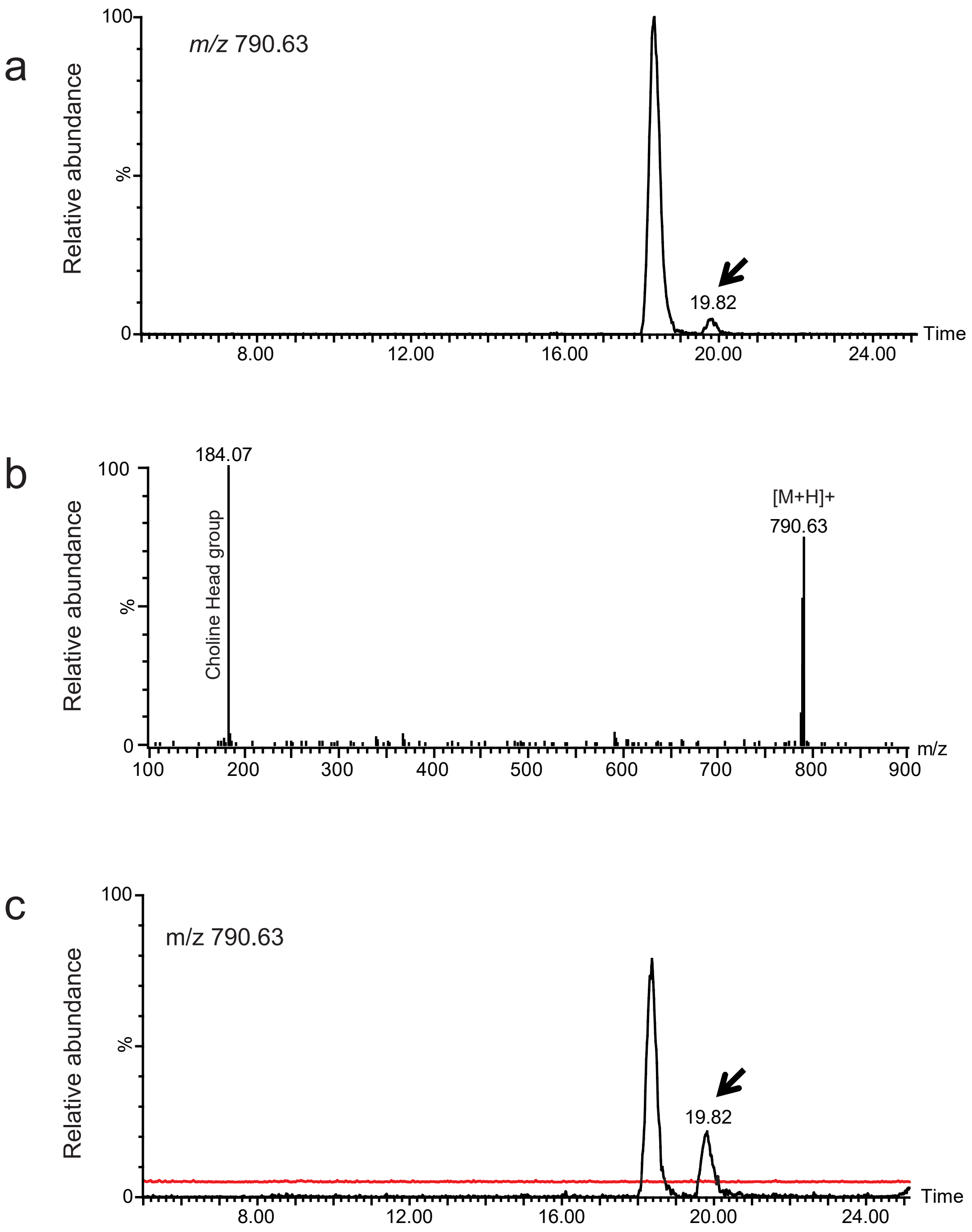

Supplementary Figure S5. Identification of PC 36:0 (*m/z* = 790.63) in diluted extract (1:50,000) of whole hippocampal tissue (A: representative nLC tracing; B: mass spectrum) and isopropanol extract of a single DG granule cell (C: representative nLC tracing). Black tracings: neu-rons; red tracings: artificial cerebrospinal fluid.

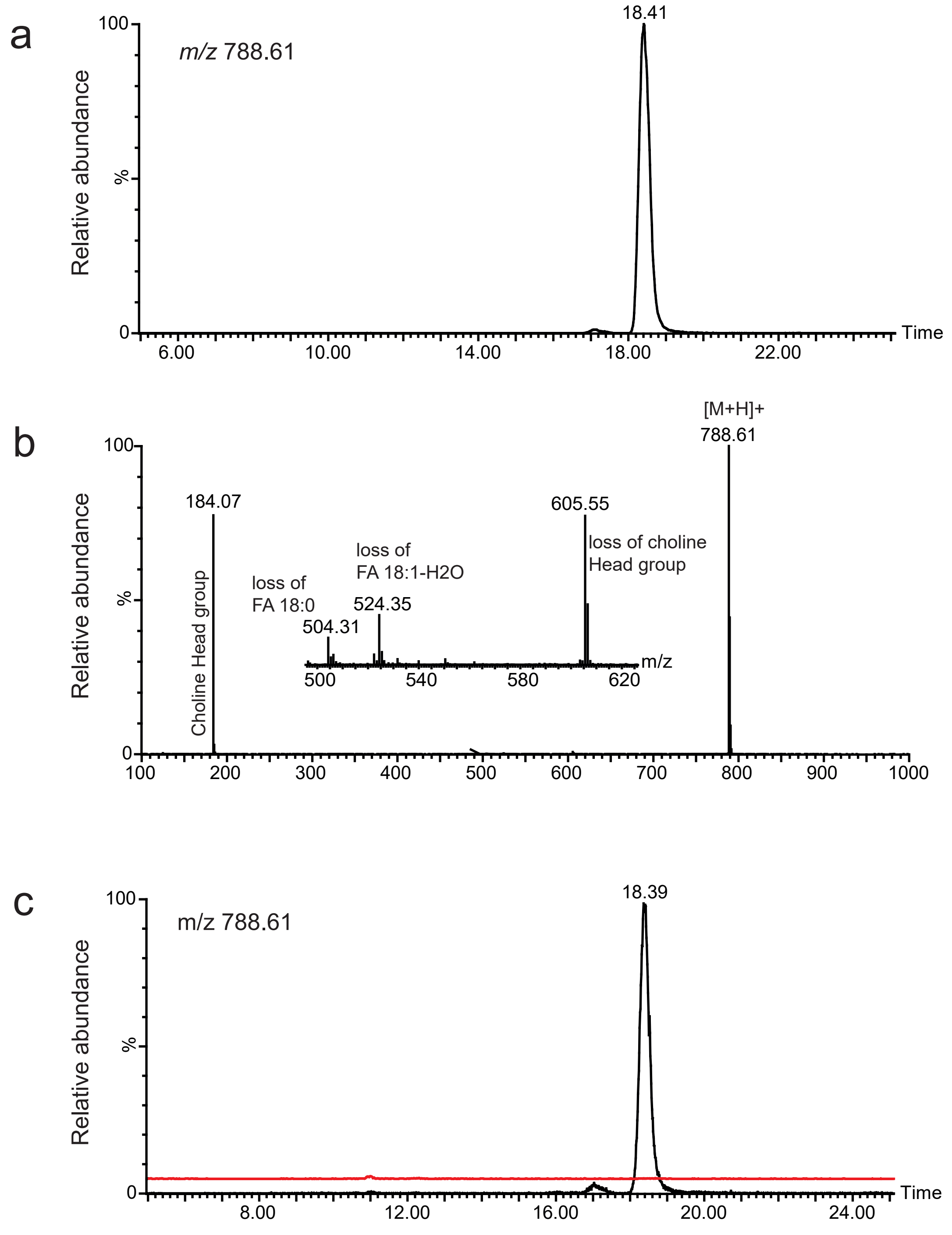

Supplementary Figure S6. Identification of PC 36:1 (*m/z* = 788.61) in diluted extract (1:50,000) of whole hippocampal tissue (A: representative nLC tracing; B: mass spectrum) and isopropanol extract of a single DG granule cell (C: representative nLC tracing). Black tracings: neurons; red tracings: artificial cerebrospinal fluid.

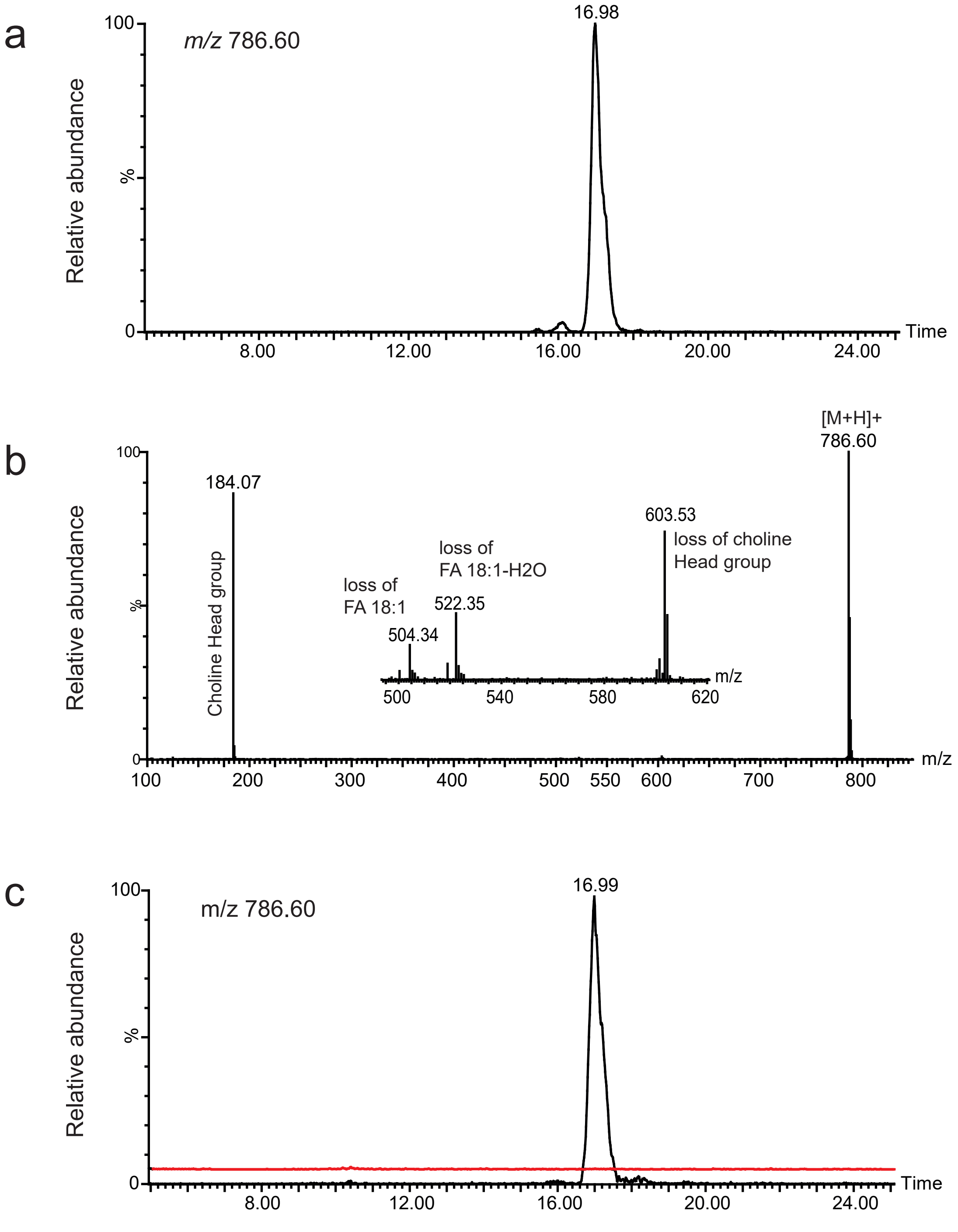

Supplementary Figure S7. Identification of PC 36:2 (*m/z* = 786.60) in diluted extract (1:50,000) of whole hippocampal tissue (A: representative nLC tracing; B: mass spectrum) and isopropanol extract of a single DG granule cell (C: representative nLC tracing). Black tracings: neurons; red tracings: artificial cerebrospinal fluid.

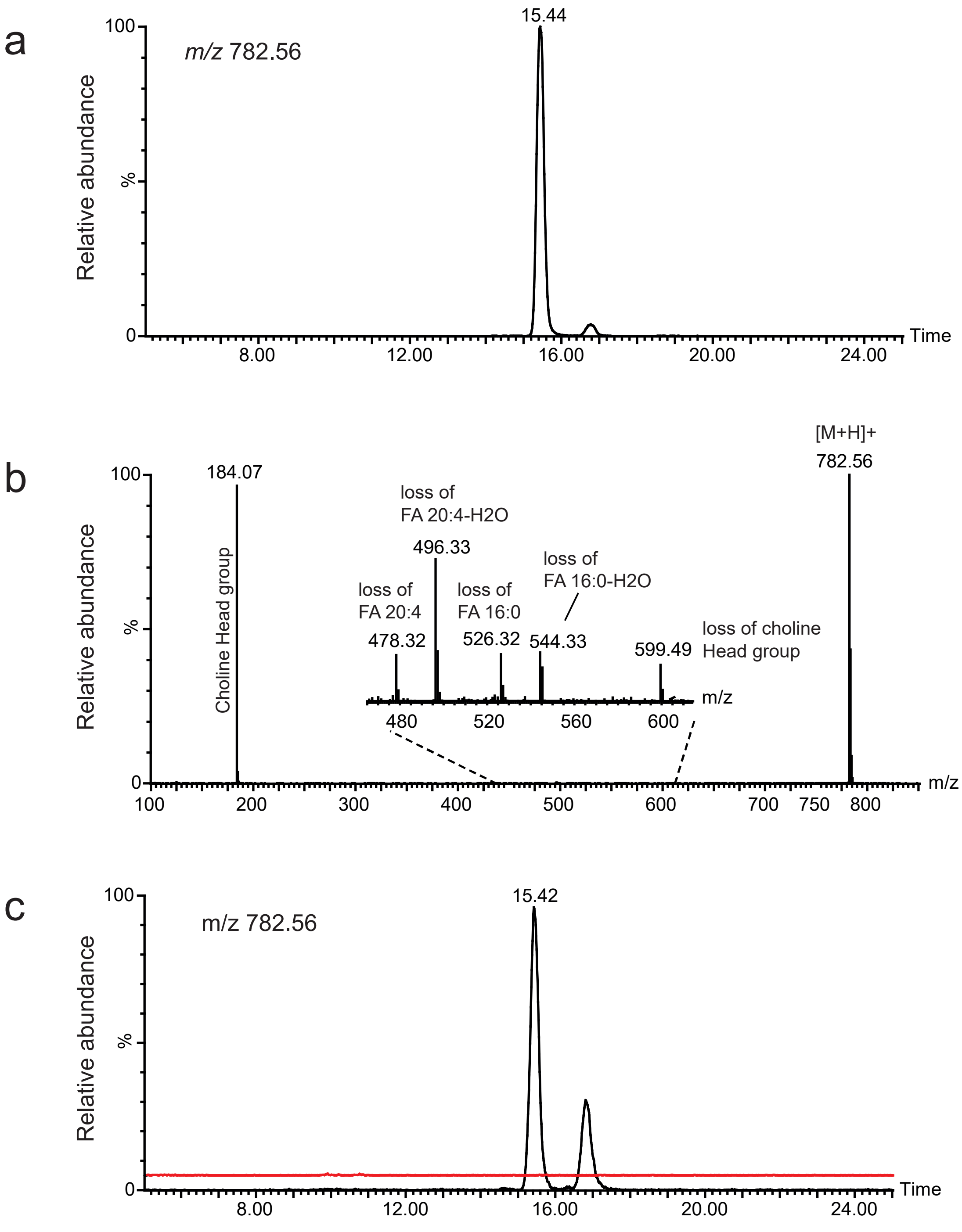

Supplementary Figure S8. Identification of PC 36:4 (*m/z* = 782.56) in diluted extract (1:50,000) of whole hippocampal tissue (A: representative nLC tracing; B: mass spectrum) and isopropanol extract of a single DG granule cell (C: representative nLC tracing). Black tracings: neurons; red tracings: artificial cerebrospinal fluid.

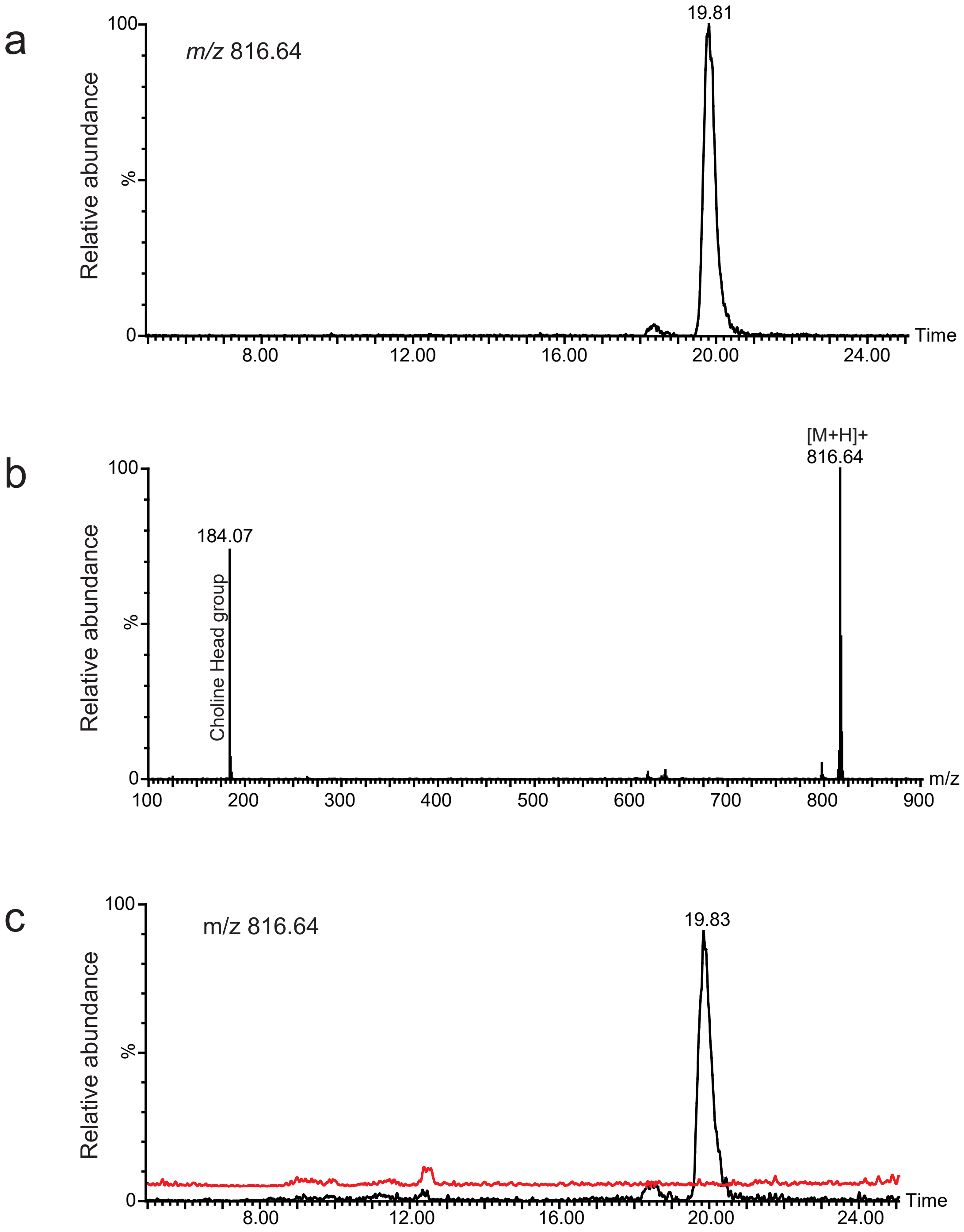

Supplementary Figure S9. Identification of PC 38:1 (*m/z* = 816.64) in diluted extract (1:50,000) of whole hippocampal tissue (A: representative nLC tracing; B: mass spectrum) and isopropanol extract of a single DG granule cell (C: representative nLC tracing). Black tracings: neurons; red tracings: artificial cerebrospinal fluid.

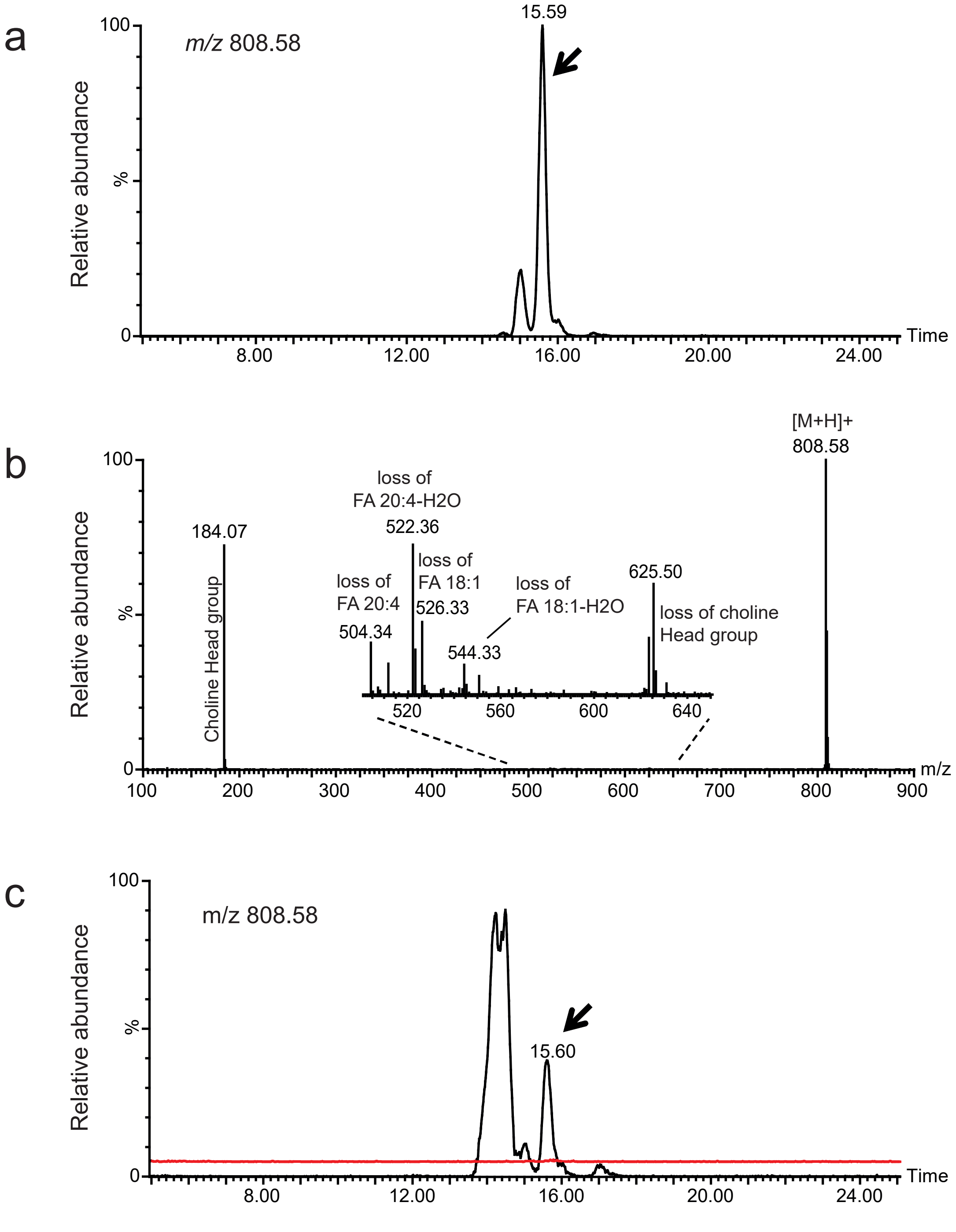

Supplementary Figure S10. Identification of PC 38:5 (*m/z* = 808.58) in diluted extract (1:50,000) of whole hippocampal tissue (A: representative nLC tracing; B: mass spectrum) and isopropanol extract of a single DG granule cell (C: representative nLC tracing). Black tracings: neurons; red tracings: artificial cerebrospinal fluid.

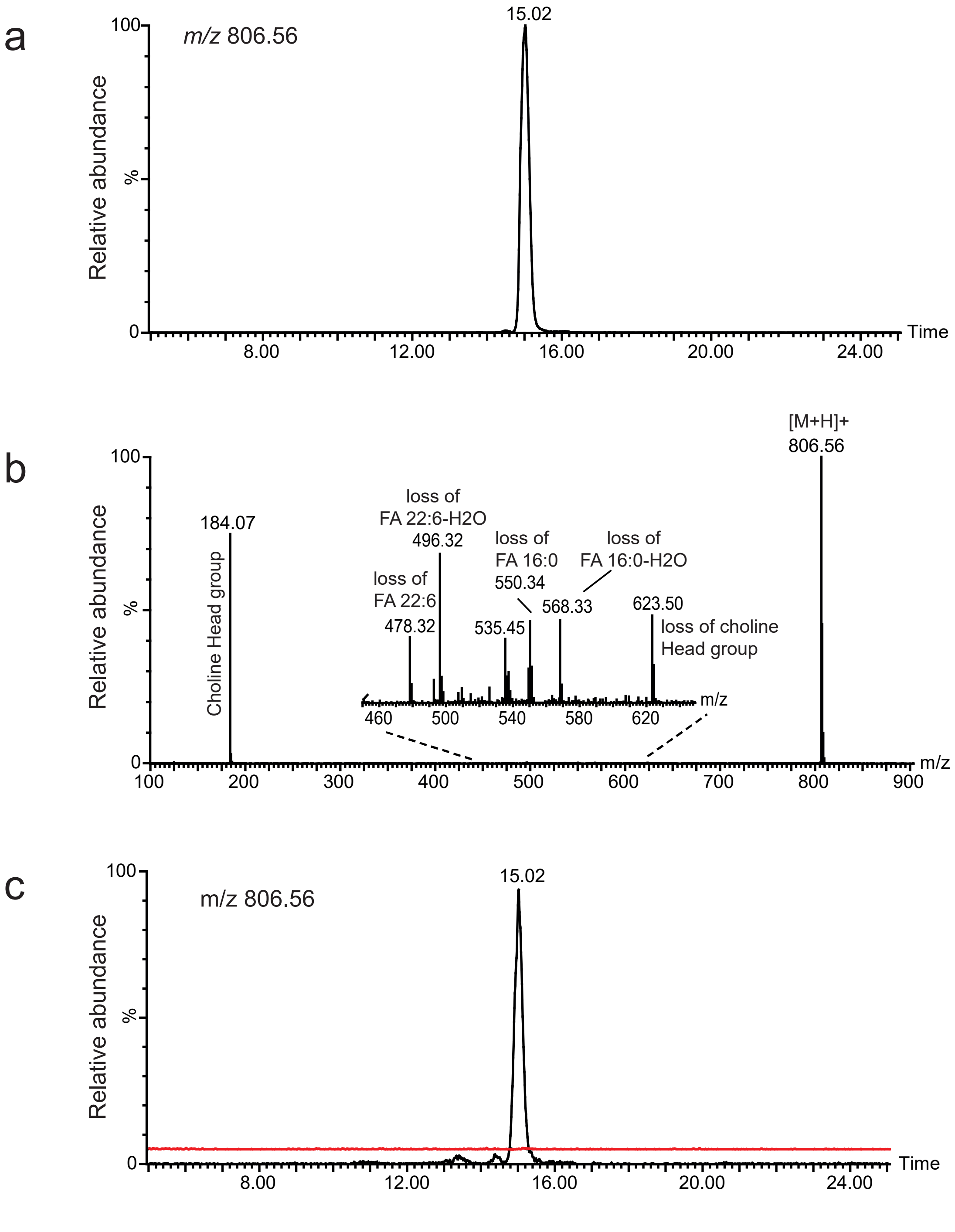

Supplementary Figure S11. Identification of PC 38:6 (*m/z* = 806.56) in diluted extract (1:50,000) of whole hippocampal tissue (A: representative nLC tracing; B: mass spectrum) and isopropanol extract of a single DG granule cell (C: representative nLC tracing). Black tracings: neurons; red tracings: artificial cerebrospinal fluid.

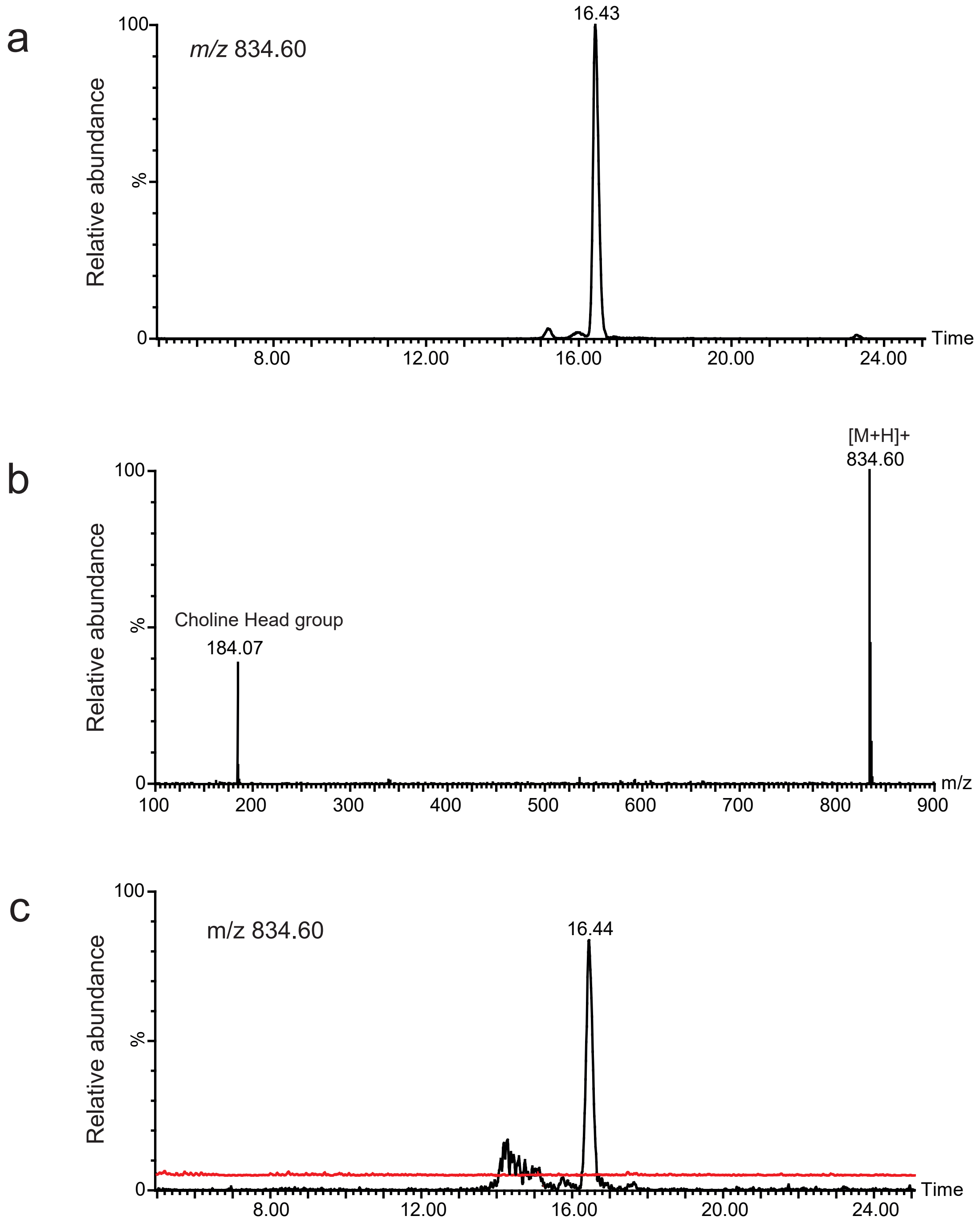

Supplementary Figure S12. Identification of PC 40:6 (*m/z* = 834.60) in diluted extract (1:50,000) of whole hippocampal tissue (A: representative nLC tracing; B: mass spectrum) and isopropanol extract of a single DG granule cell (C: representative nLC tracing). Black tracings: neurons; red tracings: artificial cerebrospinal fluid.

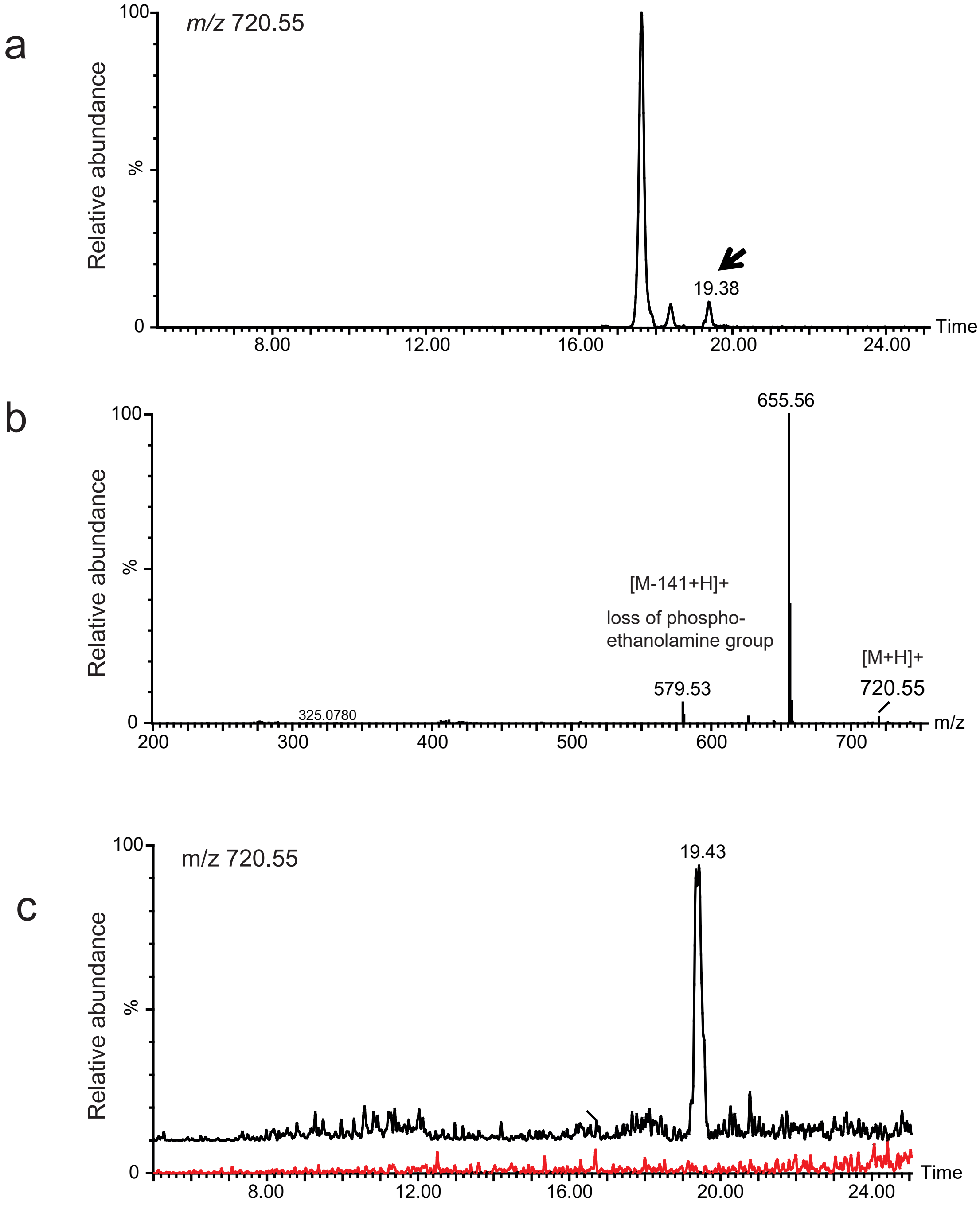

Supplementary Figure S13. Identification of PE 34:0 (*m/z* = 720.55) in diluted extract (1:50,000) of whole hippocampal tissue (A: representative nLC tracing; B: mass spectrum) and isopropanol extract of a single DG granule cell (C: representative nLC tracing). Black tracings: neurons; red tracings: artificial cerebrospinal fluid.

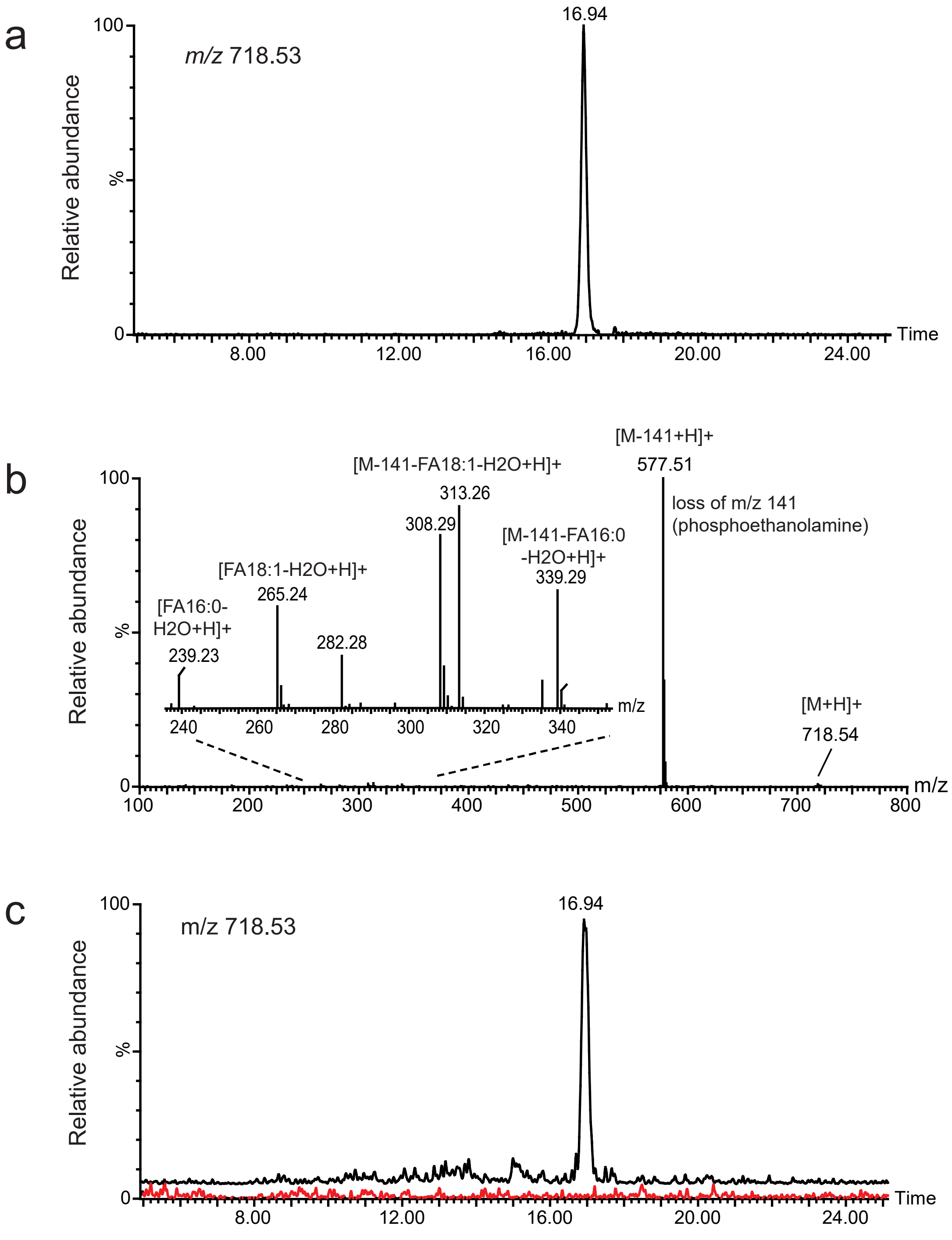

Supplementary Figure S14. Identification of PE 34:1 (*m/z* = 718.53) in diluted extract (1:50,000) of whole hippocampal tissue (A: representative nLC tracing; B: mass spectrum) and isopropanol extract of a single DG granule cell (C: representative nLC tracing). Black tracings: neurons; red tracings: artificial cerebrospinal fluid.

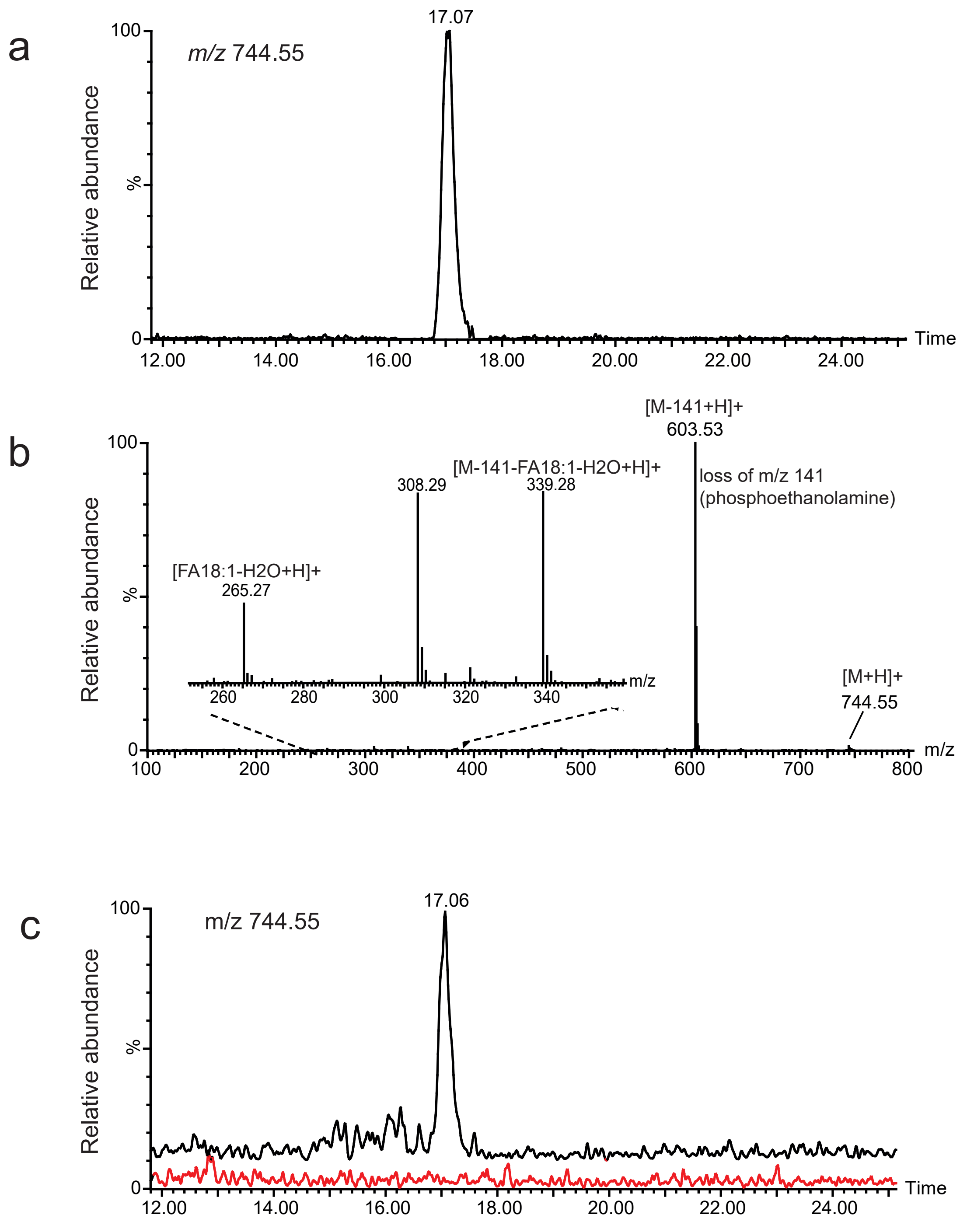

Supplementary Figure S15. Identification of PE 36:2 (*m/z* = 744.55) in diluted extract (1:50,000) of whole hippocampal tissue (A: representative nLC tracing; B: mass spectrum) and isopropanol extract of a single DG granule cell (C: representative nLC tracing). Black tracings: neurons; red tracings: artificial cerebrospinal fluid.

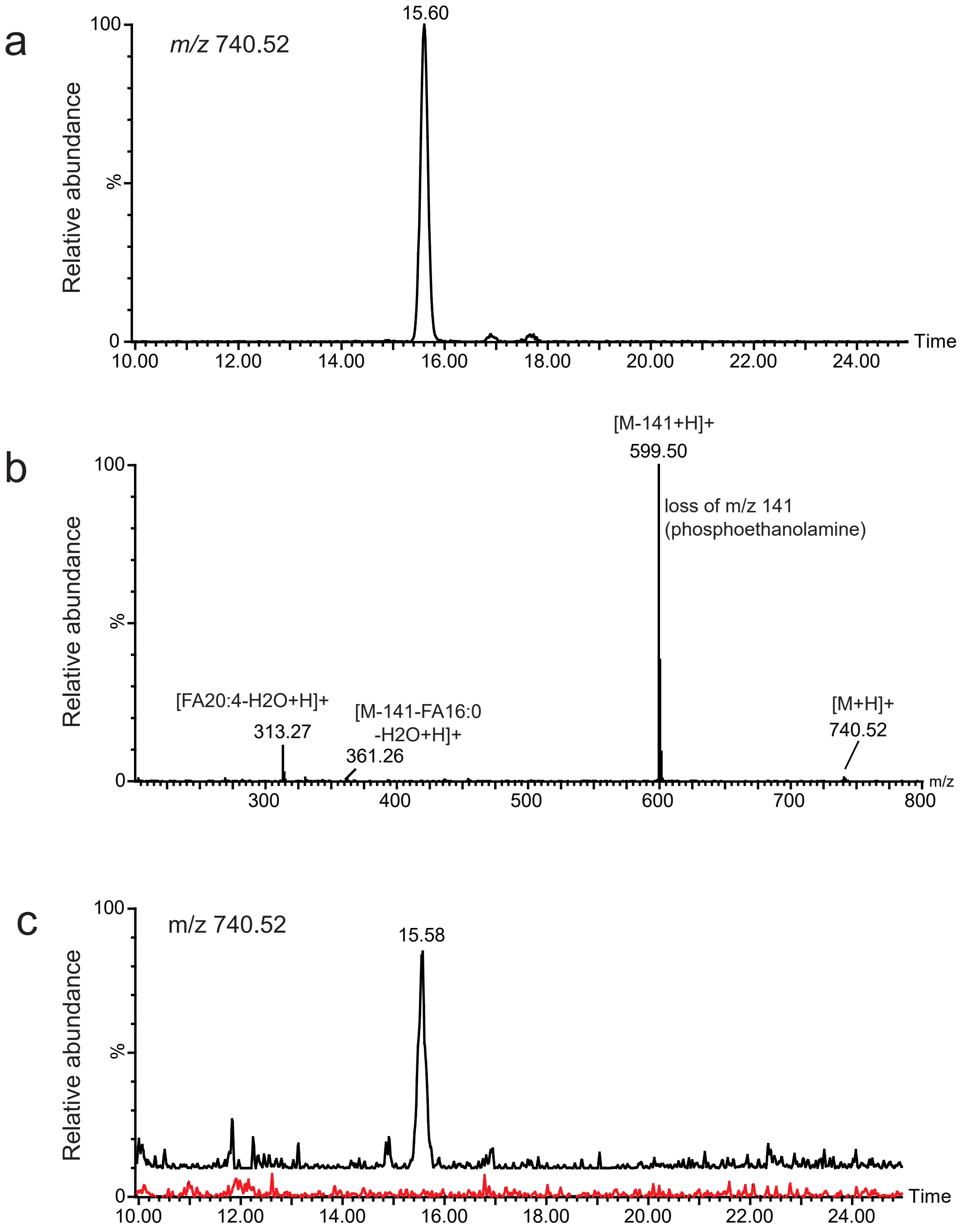

Supplementary Figure S16. Identification of PE 36:4 (*m/z* = 740.52) in diluted extract (1:50,000) of whole hippocampal tissue (A: representative nLC tracing; B: mass spectrum) and isopropanol extract of a single DG granule cell (C: representative nLC tracing). Black tracings: neurons; red tracings: artificial cerebrospinal fluid.

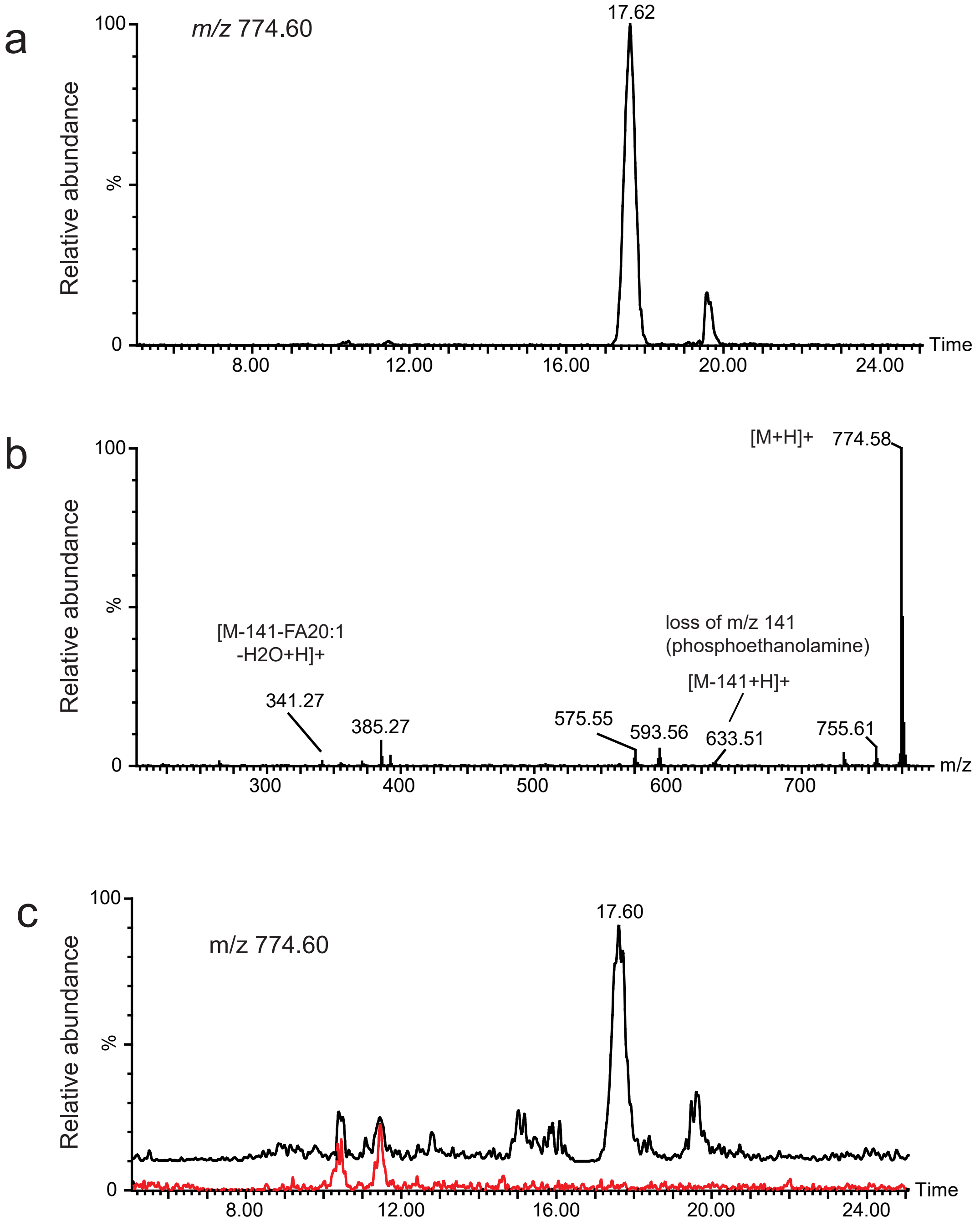

Supplementary Figure S17. Identification of PE 38:1 (*m/z* = 774.60) in diluted extract (1:50,000) of whole hippocampal tissue (A: representative nLC tracing; B: mass spectrum) and isopropanol extract of a single DG granule cell (C: representative nLC tracing). Black tracings: neurons; red tracings: artificial cerebrospinal fluid.

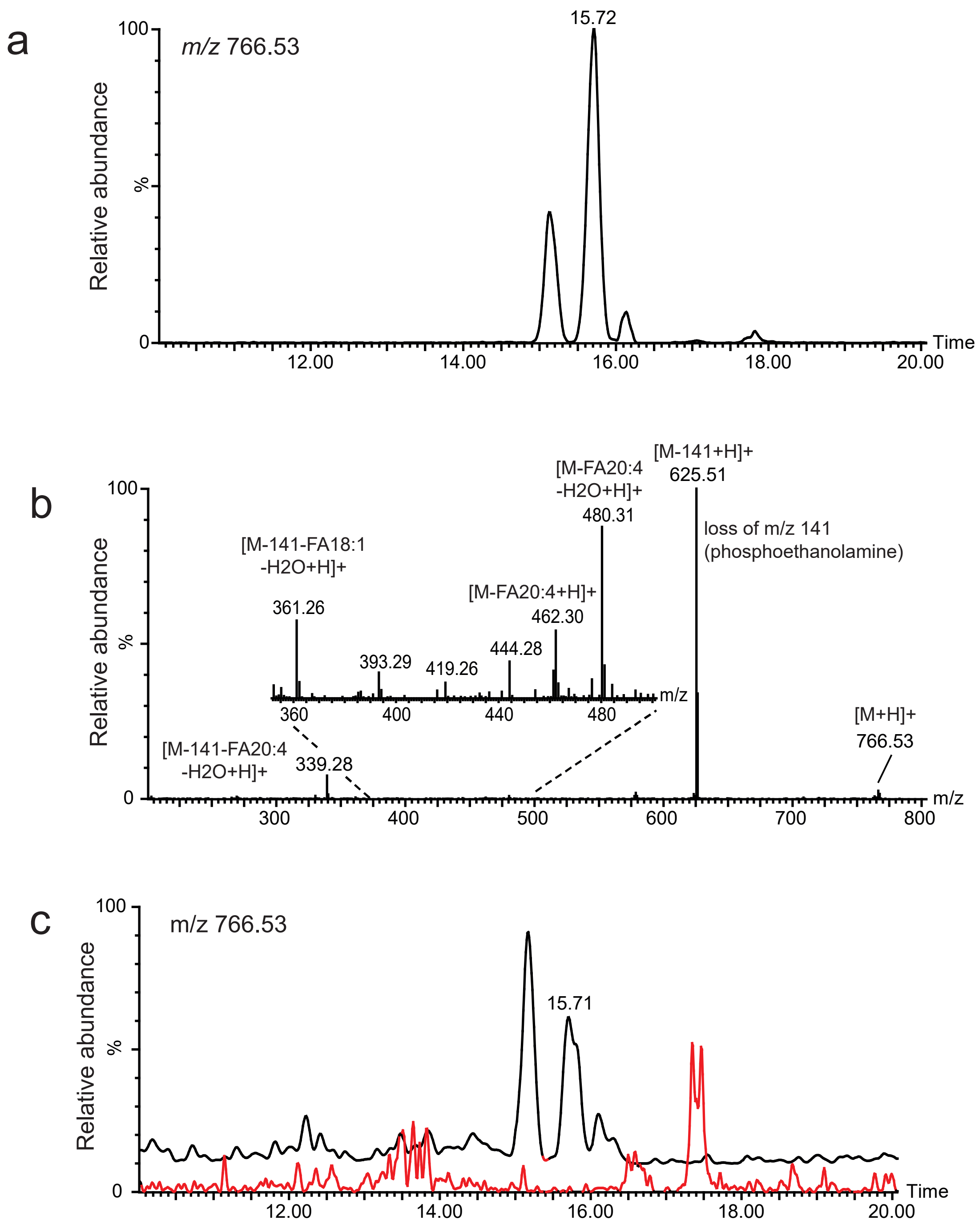

Supplementary Figure S18. Identification of PE 38:5 (*m/z* = 766.53) in diluted extract (1:50,000) of whole hippocampal tissue (A: representative nLC tracing; B: mass spectrum) and isopropanol extract of a single DG granule cell (C: representative nLC tracing). Black tracings: neurons; red tracings: artificial cerebrospinal fluid.

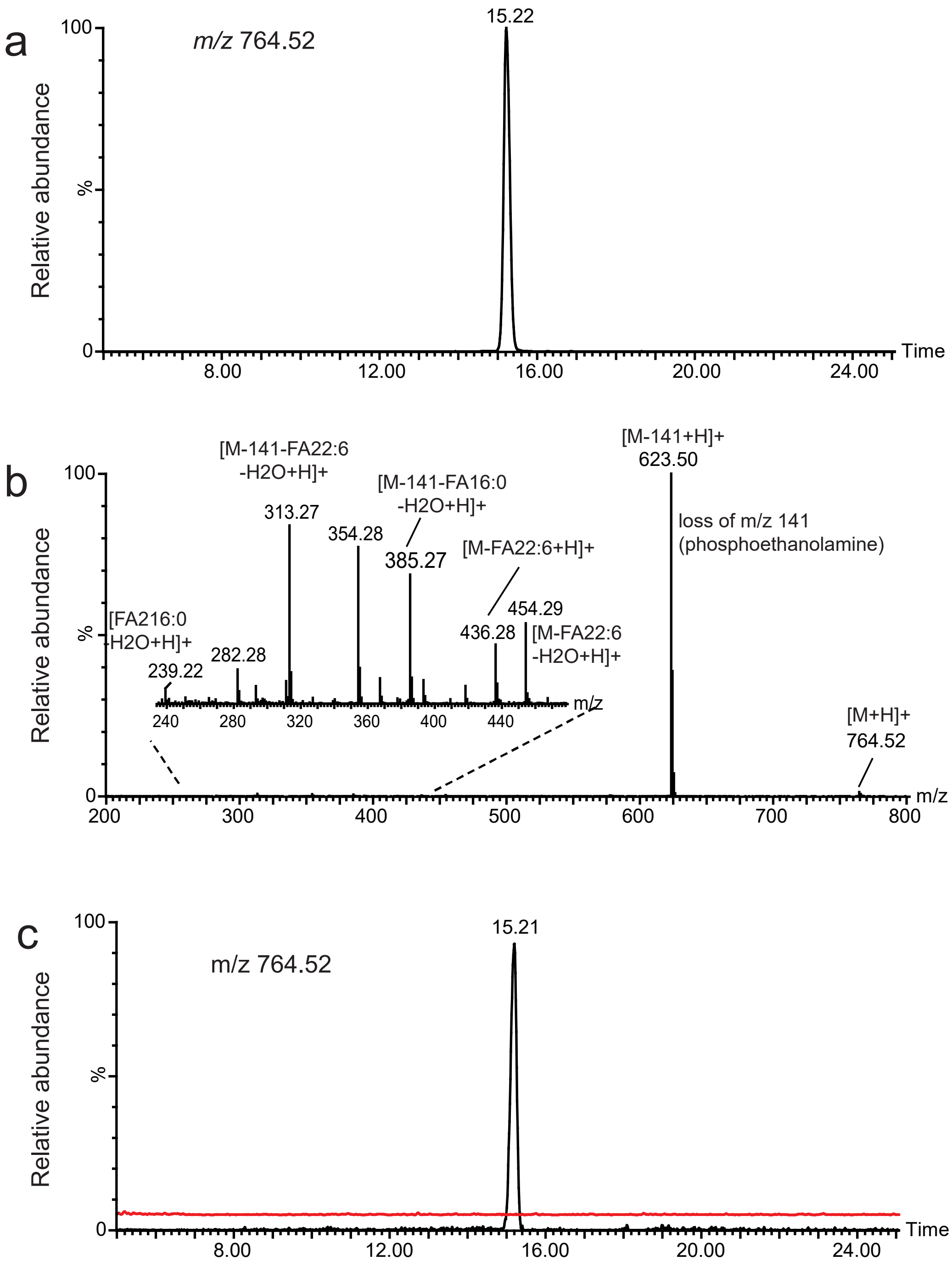

Supplementary Figure S19. Identification of PE 38:6 (*m/z* = 764.52) in diluted extract (1:50,000) of whole hippocampal tissue (A: representative nLC tracing; B: mass spectrum) and isopropanol extract of a single DG granule cell (C: representative nLC tracing). Black tracings: neurons; red tracings: artificial cerebrospinal fluid.

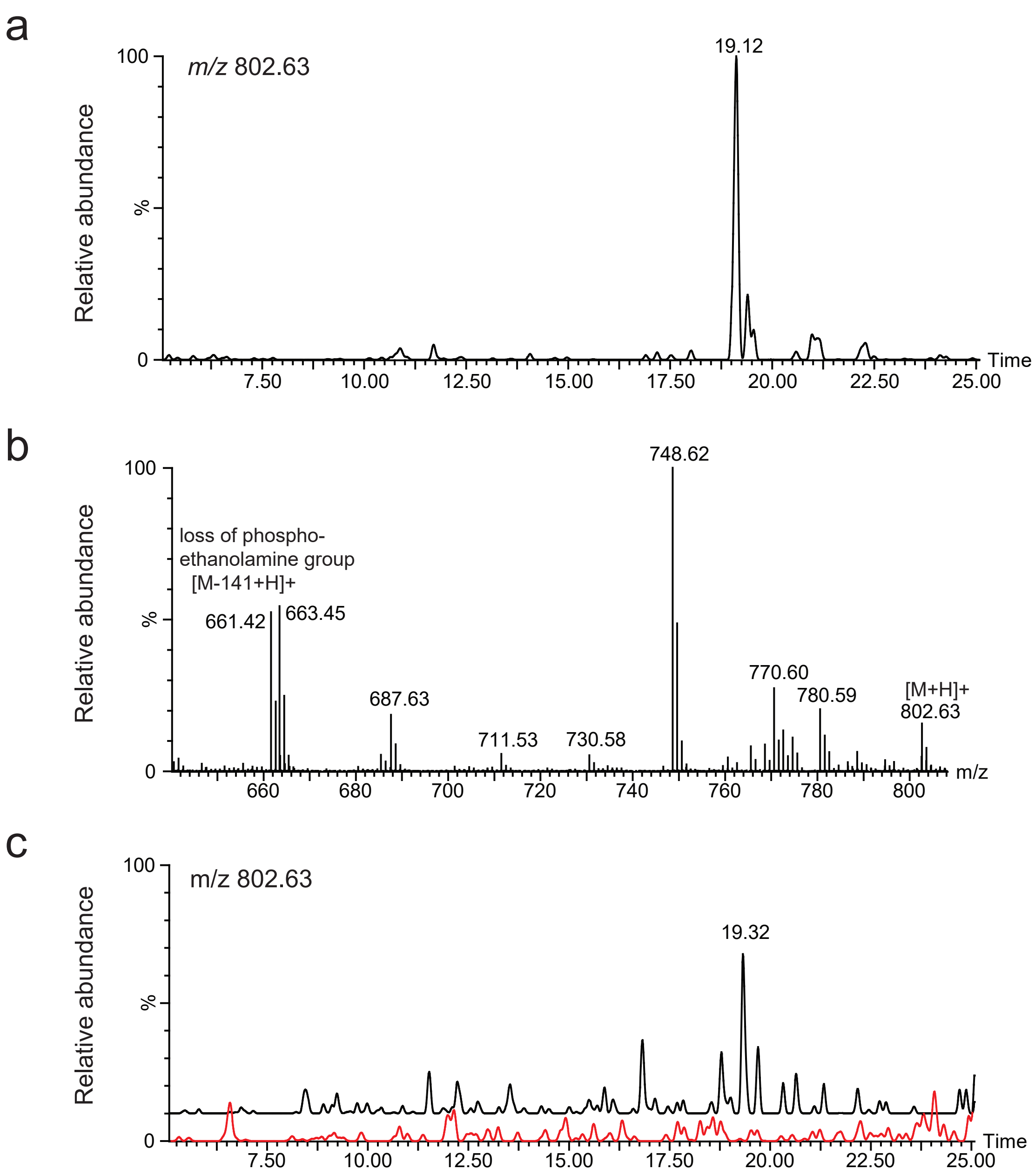

Supplementary Figure S20. Identification of PE 40:1 (*m/z* = 802.63) in diluted extract (1:50,000) of whole hippocampal tissue (A: representative nLC tracing; B: mass spectrum) and isopropanol extract of a single DG granule cell (C: representative nLC tracing). Black tracings: neurons; red tracings: artificial cerebrospinal fluid.

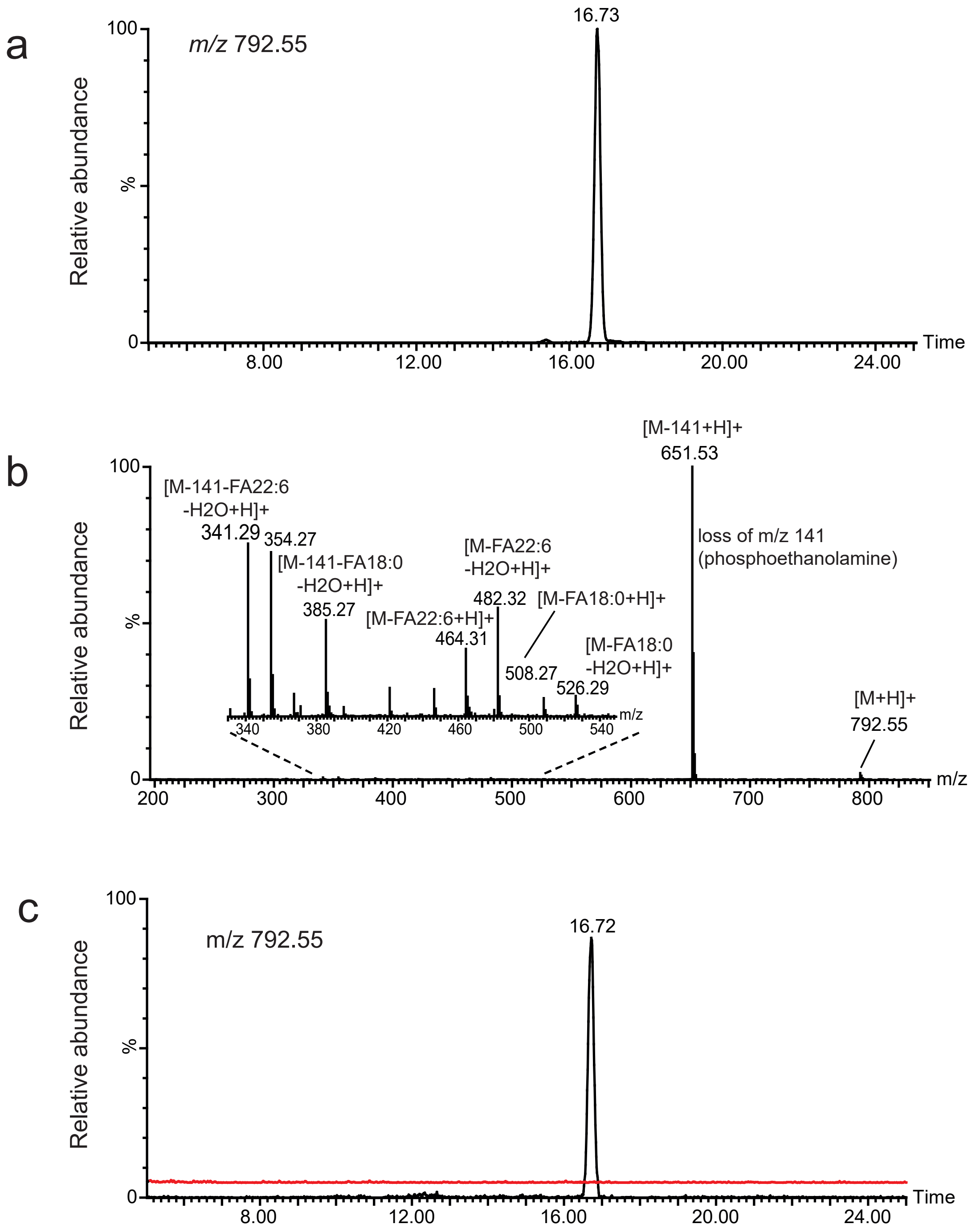

Supplementary Figure S21. Identification of PE 40:6 (*m/z* = 792.55) in diluted extract (1:50,000) of whole hippocampal tissue (A: representative nLC tracing; B: mass spectrum) and isopropanol extract of a single DG granule cell (C: representative nLC tracing). Black tracings: neurons; red tracings: artificial cerebrospinal fluid.

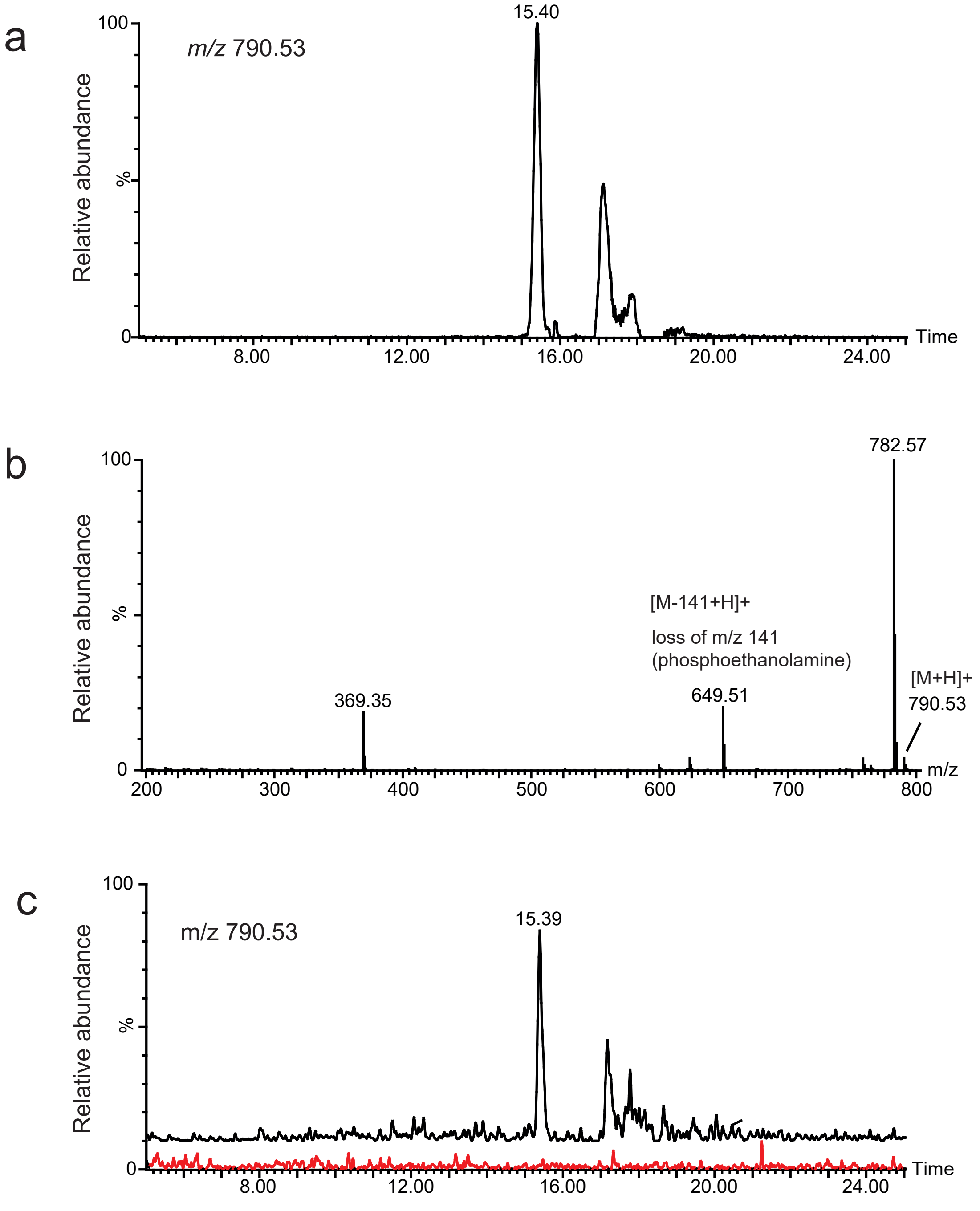

Supplementary Figure S22. Identification of PE 40:7 (*m/z* = 790.53) in diluted extract (1:50,000) of whole hippocampal tissue (A: representative nLC tracing; B: mass spectrum) and isopropanol extract of a single DG granule cell (C: representative nLC tracing). Black tracings: neurons; red tracings: artificial cerebrospinal fluid.

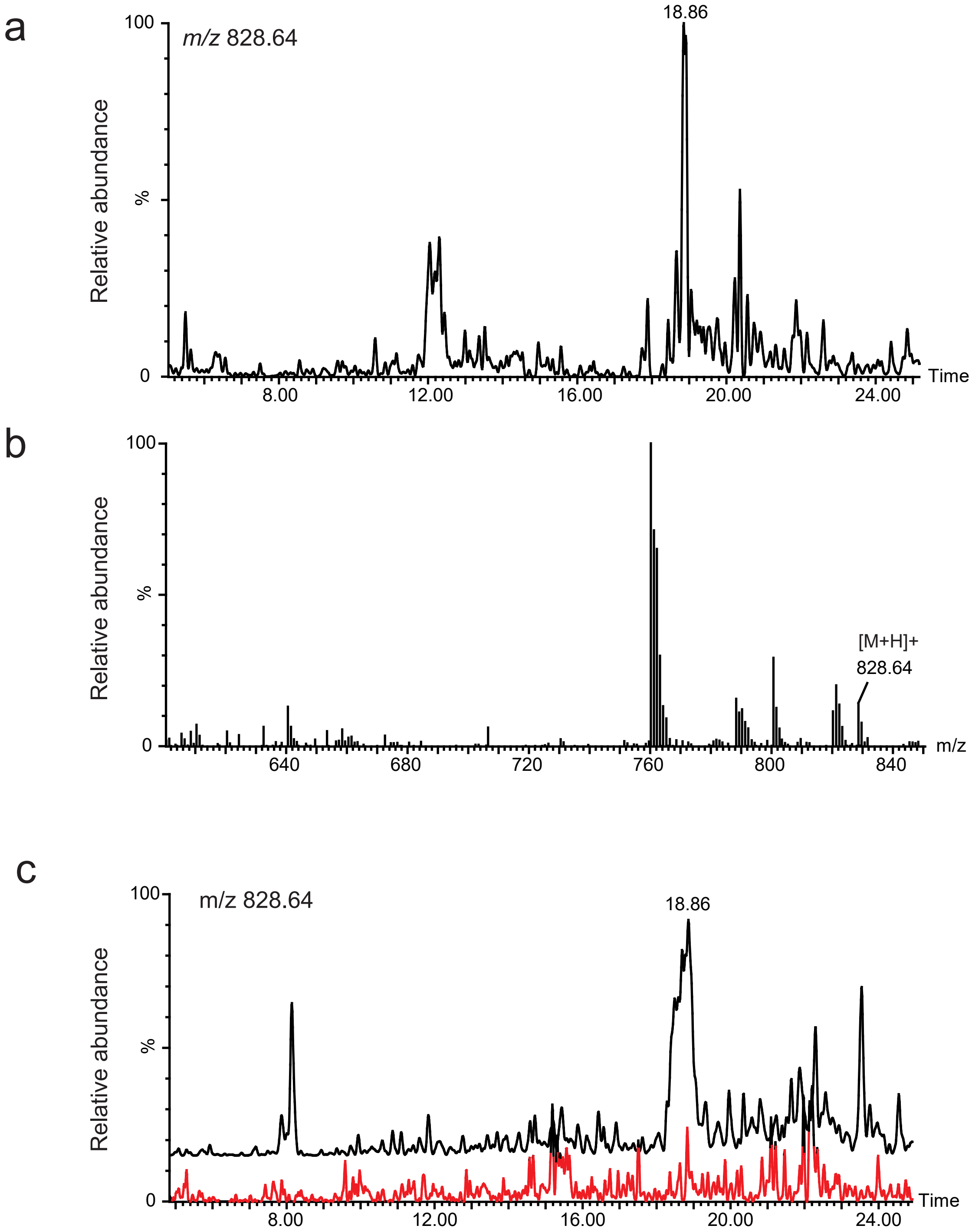

Supplementary Figure S23. Identification of PE 42:2 (*m/z* = 828.64) in diluted extract (1:50,000) of whole hippocampal tissue (A: representative nLC tracing; B: mass spectrum) and isopropanol extract of a single DG granule cell (C: representative nLC tracing). Black tracings: neurons; red tracings: artificial cerebrospinal fluid.

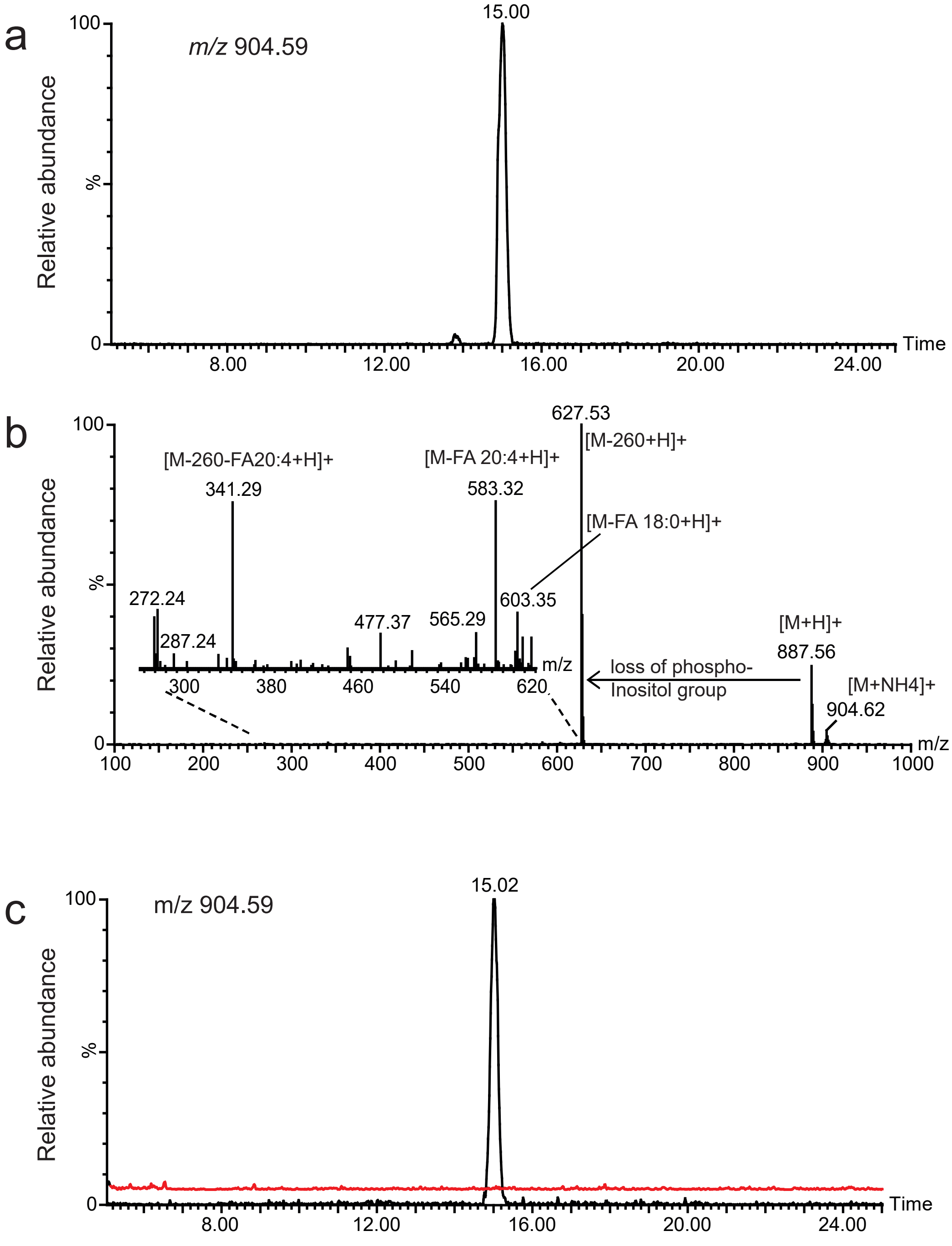

Supplementary Figure S24. Identification of PI 38:4 (*m/z* = 904.59) in diluted extract (1:50,000) of whole hippocampal tissue (A: representative nLC tracing; B: mass spectrum) and isopropanol extract of a single DG granule cell (C: representative nLC tracing). Black tracings: neurons; red tracings: artificial cerebrospinal fluid.

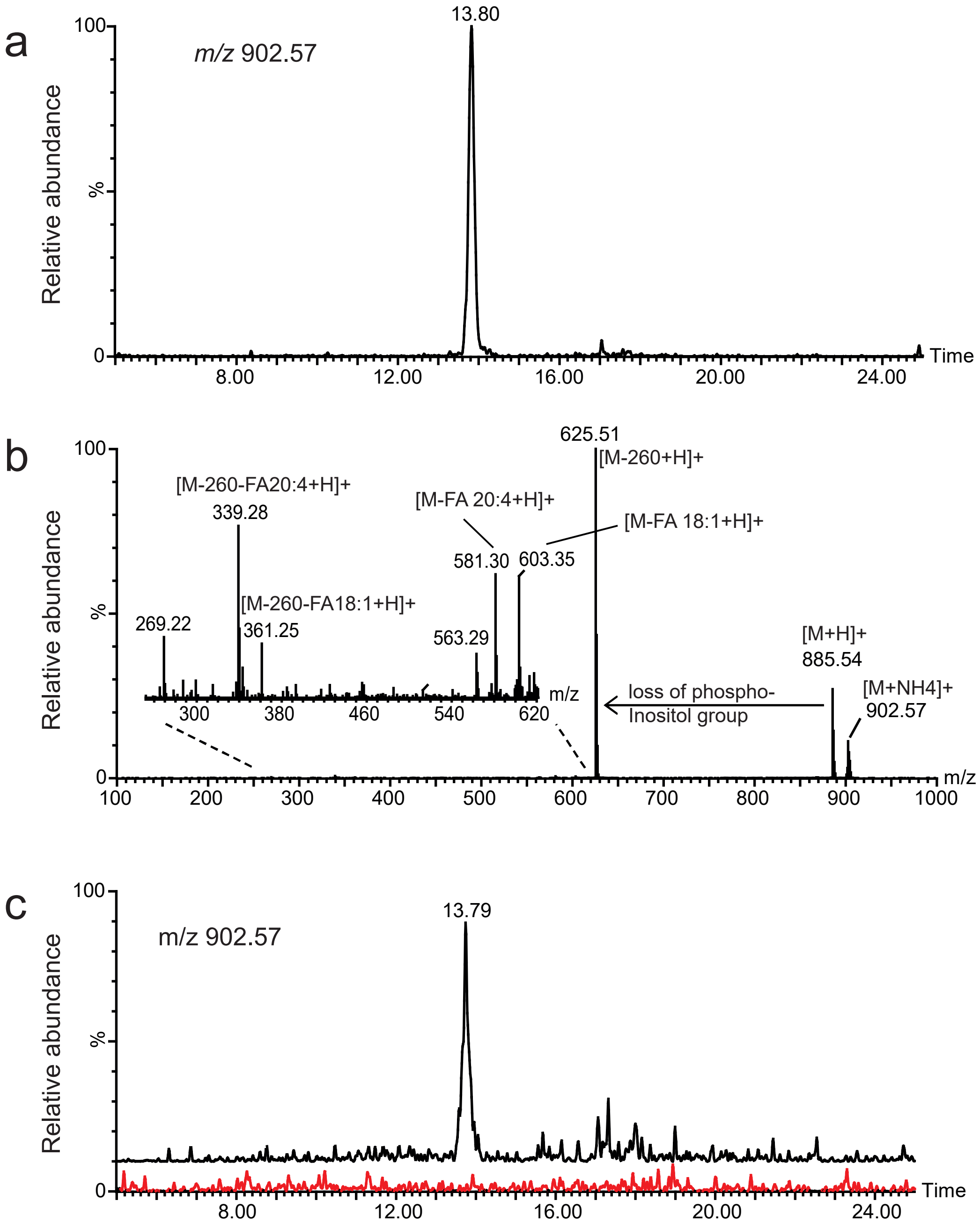

Supplementary Figure S25. Identification of PI 38:5 (*m/z* = 902.57) in diluted extract (1:50,000) of whole hippocampal tissue (A: representative nLC tracing; B: mass spectrum) and isopropanol extract of a single DG granule cell (C: representative nLC tracing). Black tracings: neurons; red tracings: artificial cerebrospinal fluid.

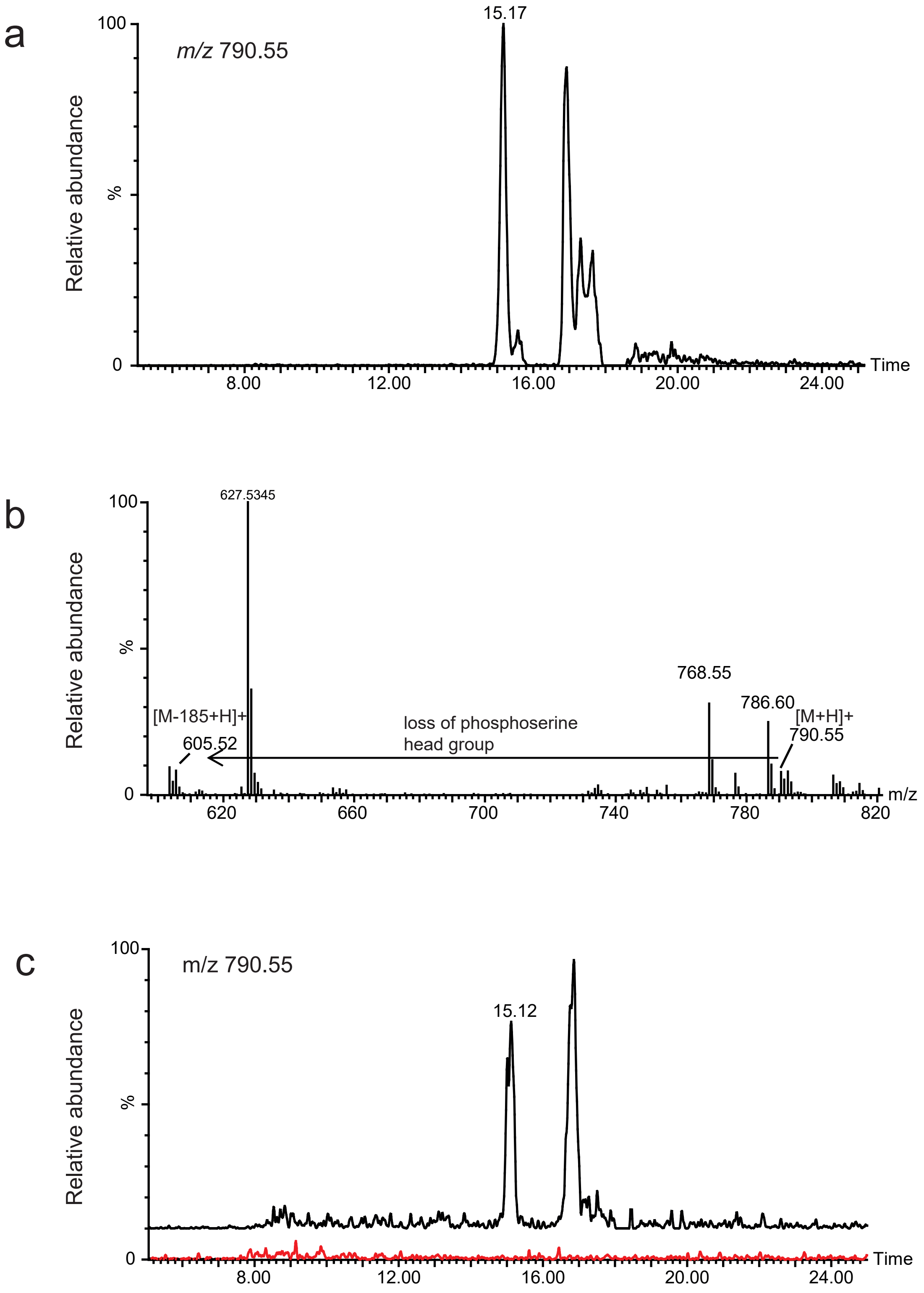

Supplementary Figure S26. Identification of PS 36:1 (*m/z* = 790.55) in diluted extract (1:50,000) of whole hippocampal tissue (A: representative nLC tracing; B: mass spectrum) and isopropanol extract of a single DG granule cell (C: representative nLC tracing). Black tracings: neurons; red tracings: artificial cerebrospinal fluid.

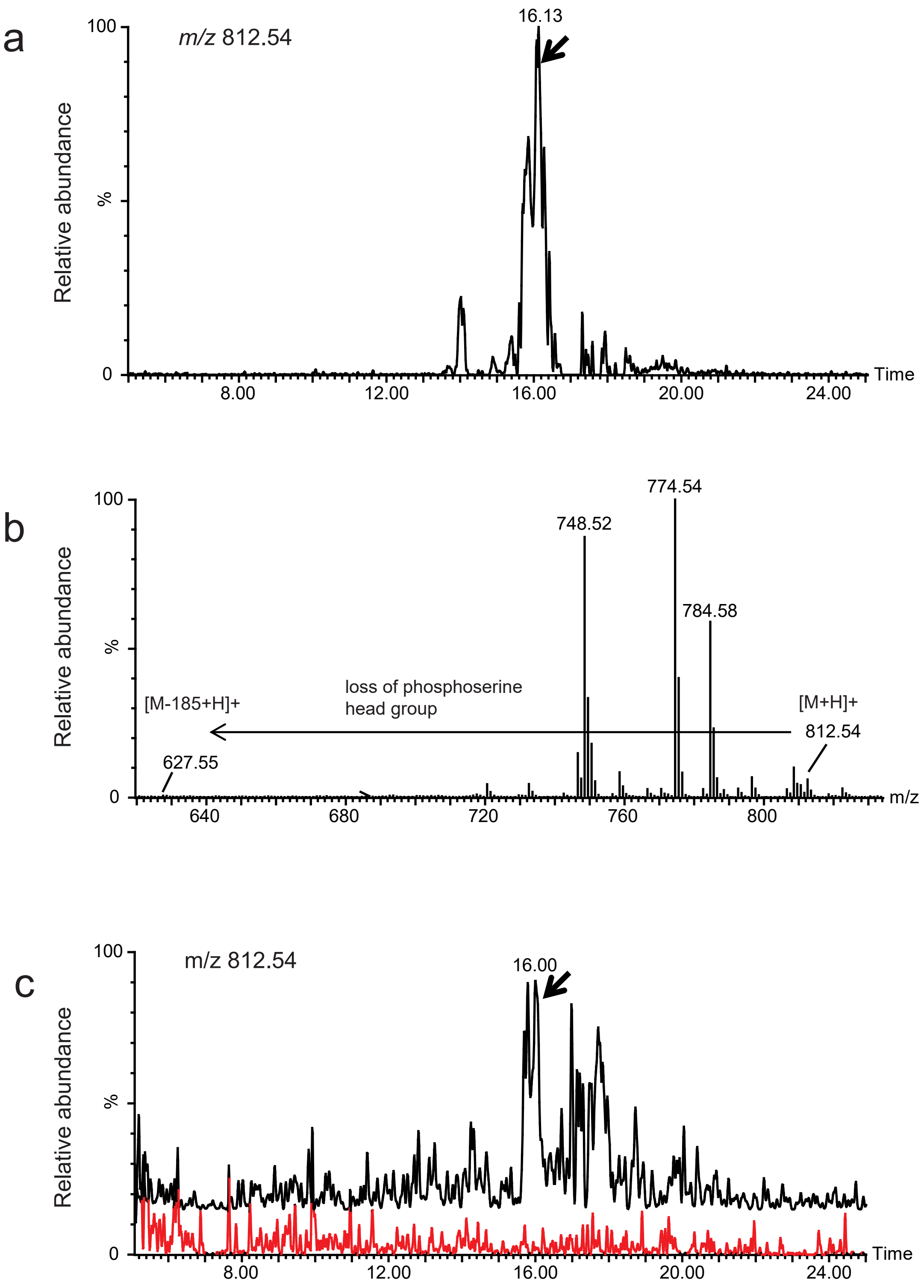

Supplementary Figure S27. Identification of PS 38:4 (*m/z* = 812.54) in diluted extract (1:50,000) of whole hippocampal tissue (A: representative nLC tracing; B: mass spectrum) and isopropanol extract of a single DG granule cell (C: representative nLC tracing). Black tracings: neurons; red tracings: artificial cerebrospinal fluid.

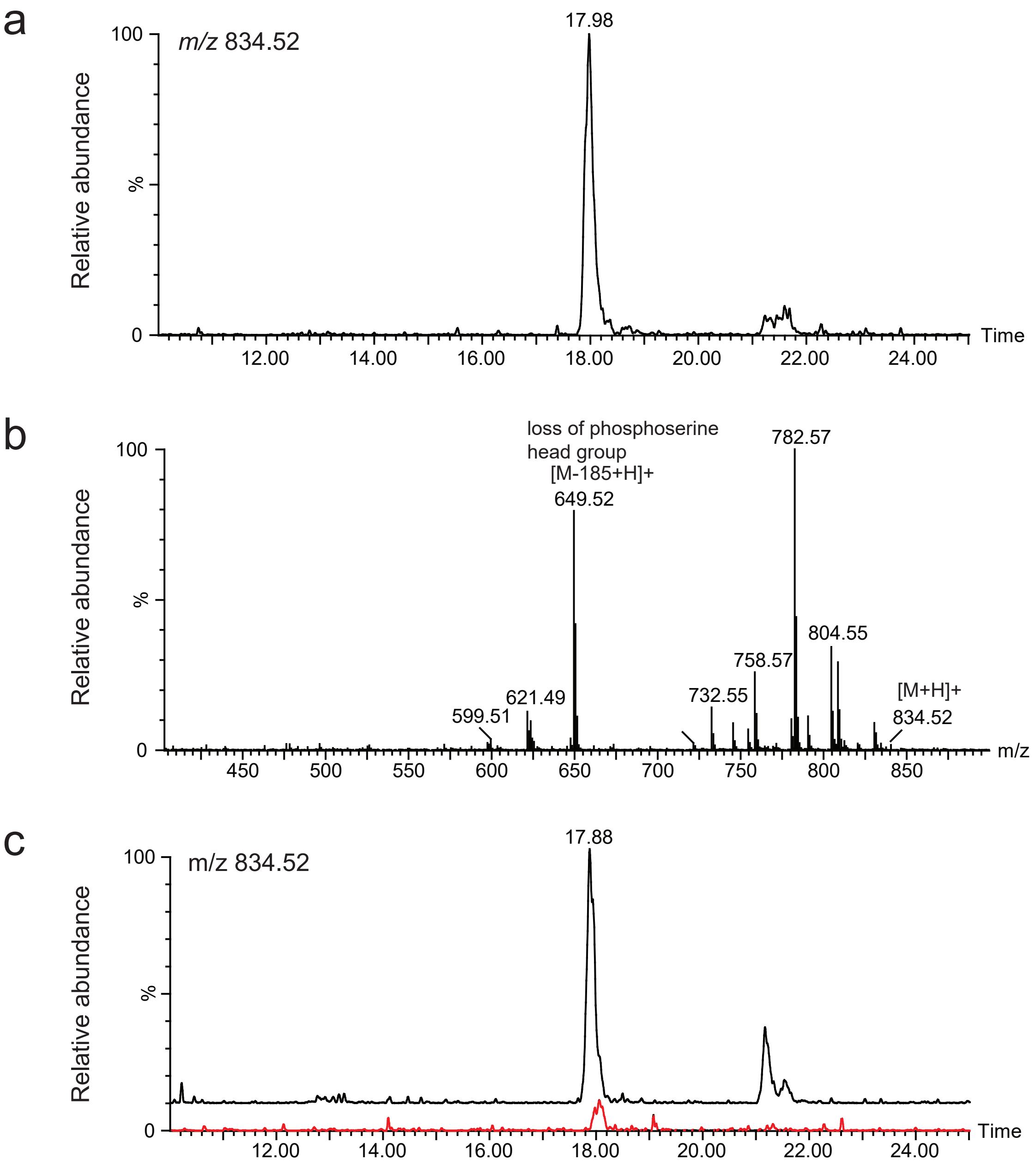

Supplementary Figure S28. Identification of PS 40:7 (*m/z* = 834.52) in diluted extract (1:50,000) of whole hippocampal tissue (A: representative nLC tracing; B: mass spectrum) and isopropanol extract of a single DG granule cell (C: representative nLC tracing). Black tracings: neurons; red tracings: artificial cerebrospinal fluid.

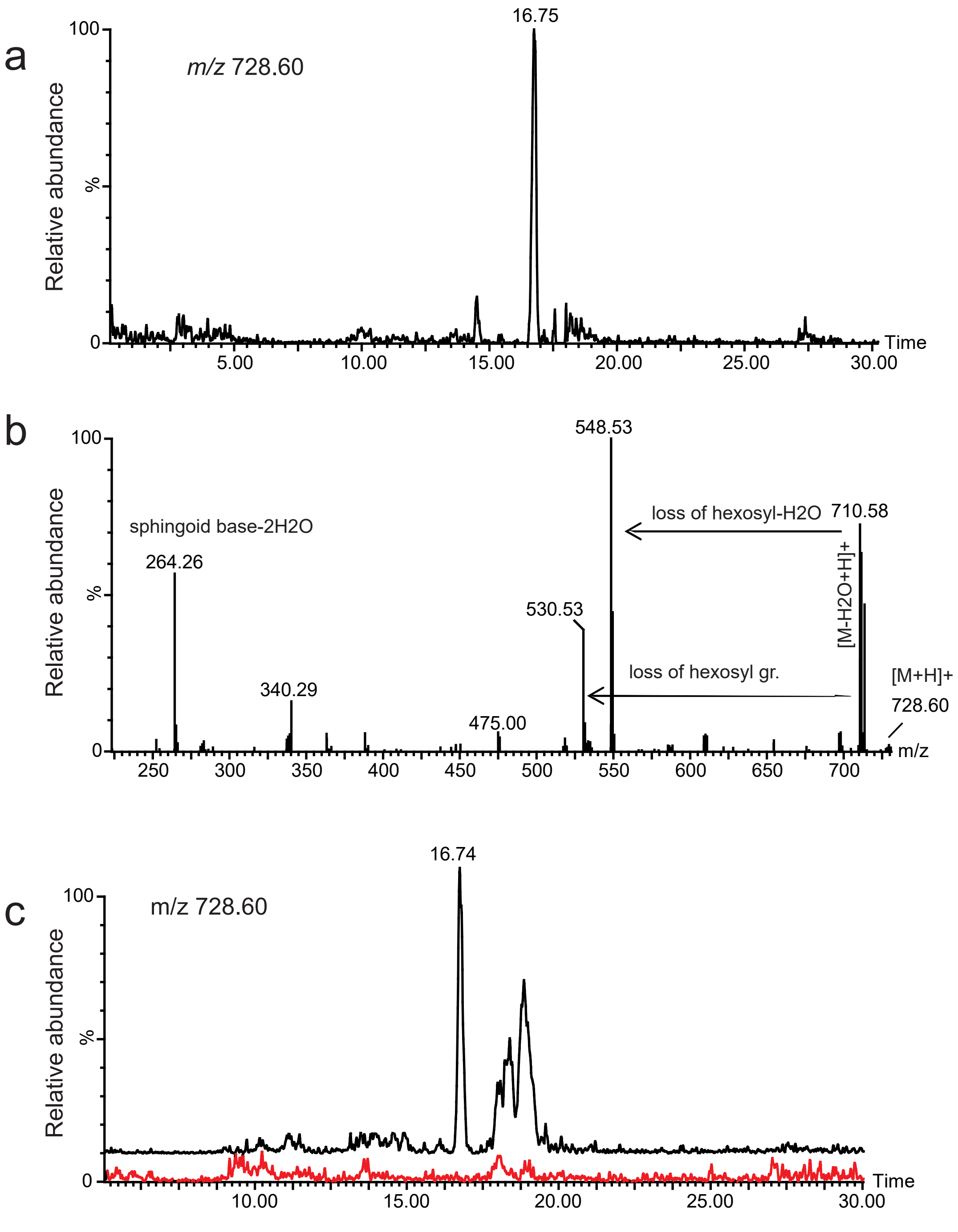

Supplementary Figure S29. Identification of HexCer (d18:1/18:0) (*m/z* = 728.60) in diluted extract (1:50,000) of whole hippocampal tissue (A: representative nLC tracing; B: mass spectrum) and isopropanol extract of a single DG granule cell (C: representative nLC tracing). Black tracings: neurons; red tracings: artificial cerebrospinal fluid.

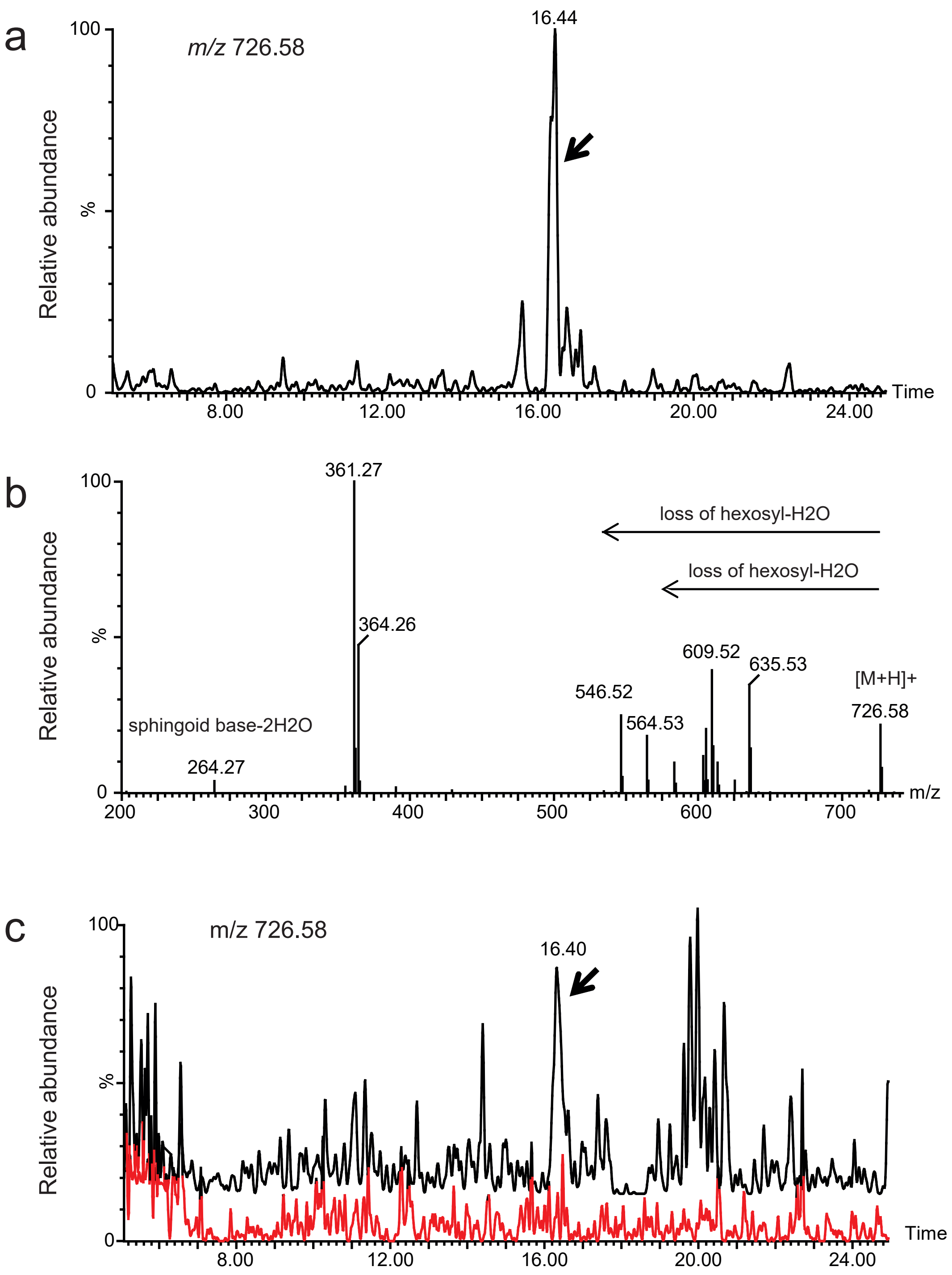

Supplementary Figure S30. Identification of HexCer (d18:1/18:1) (*m/z* = 726.58) in diluted extract (1:50,000) of whole hippocampal tissue (A: representative nLC tracing; B: mass spectrum) and isopropanol extract of a single DG granule cell (C: representative nLC tracing). Black tracings: neurons; red tracings: artificial cerebrospinal fluid.

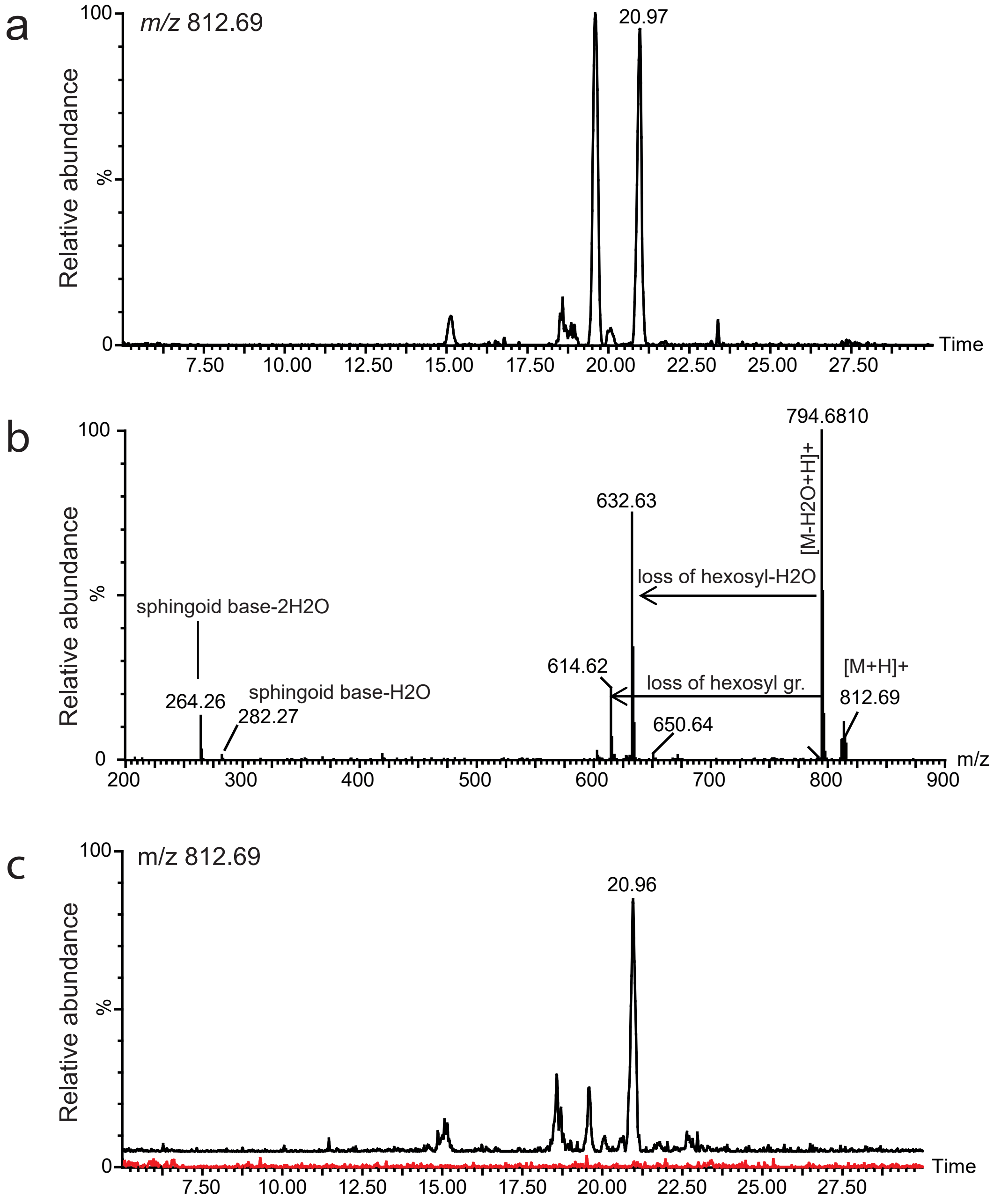

Supplementary Figure S31. Identification of HexCer (d18:1/24:0) (*m/z* = 812.69) in diluted extract (1:50,000) of whole hippocampal tissue (A: representative nLC tracing; B: mass spectrum) and isopropanol extract of a single DG granule cell (C: representative nLC tracing). Black tracings: neurons; red tracings: artificial cerebrospinal fluid.

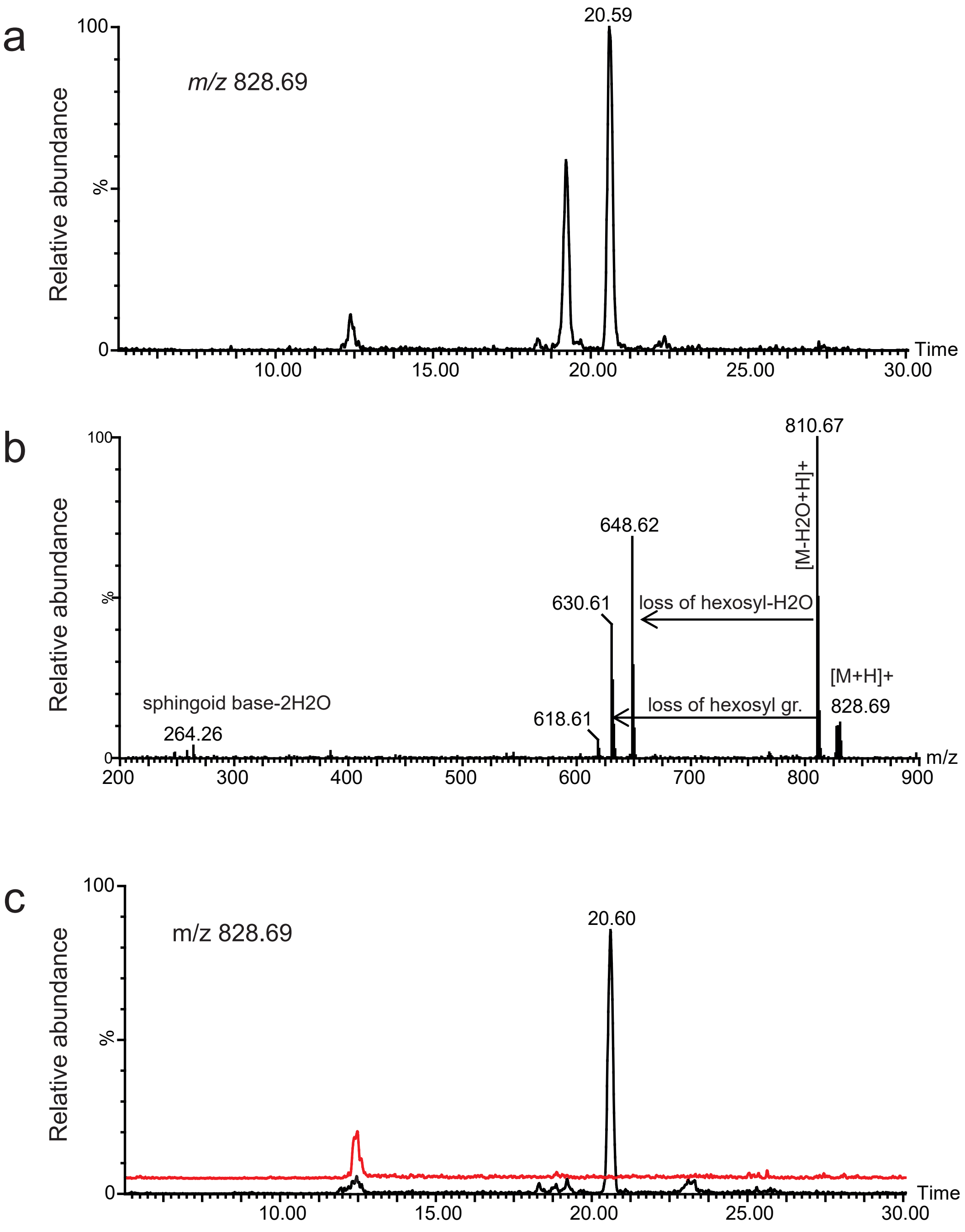

Supplemental Figure 32. Identification of HexCer (d18:1/24:0-OH) (m/z = 828.69) in diluted extract (1:50,000) of whole hippocampal tissue (A: representative nLC tracing; B: mass spectrum) and isopropanol extract of a single DG granule cell (C: representative nLC tracing). Black tracings: neurons; red tracings: artificial cerebrospinal fluid.

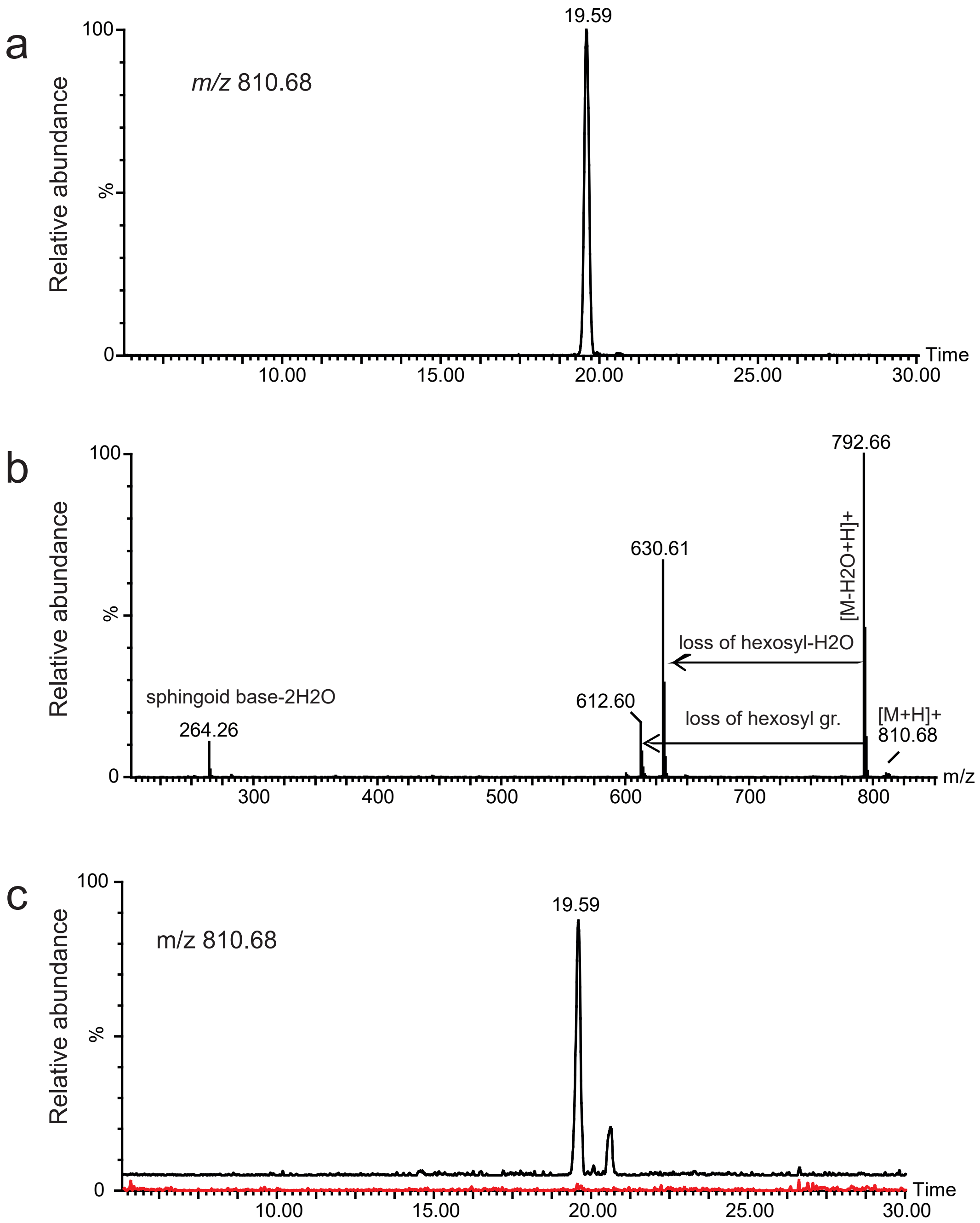

Supplementary Figure S33. Identification of HexCer (d18:1/24:1) (*m/z* = 810.68) in diluted extract (1:50,000) of whole hippocampal tissue (A: representative nLC tracing; B: mass spectrum) and isopropanol extract of a single DG granule cell (C: representative nLC tracing). Black tracings: neurons; red tracings: artificial cerebrospinal fluid.

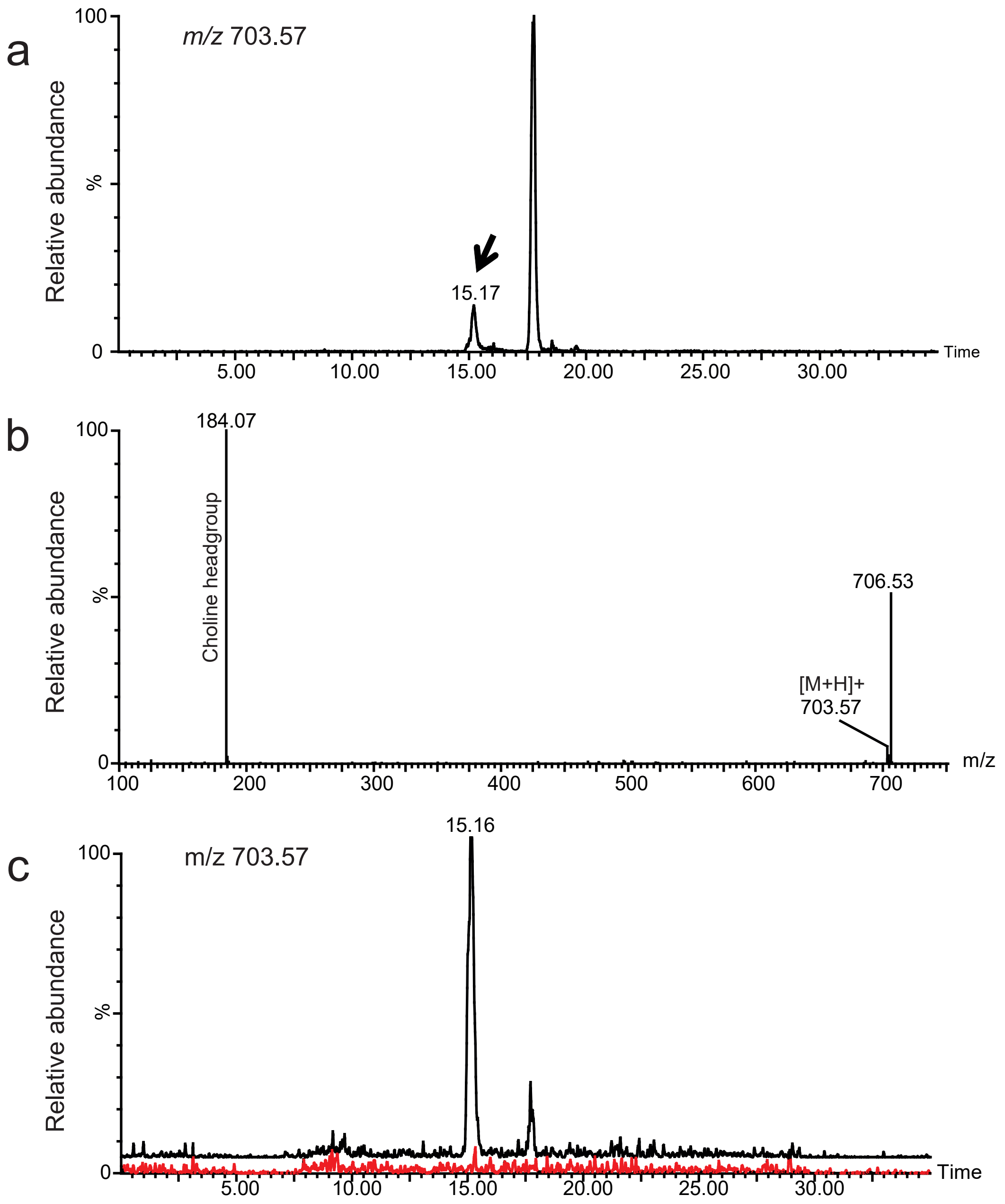

Supplementary Figure S34. Identification of SM (d18:1/16:0) (*m/z* = 703.57) in diluted extract (1:50,000) of whole hippocampal tissue (A: representative nLC tracing; B: mass spectrum) and isopropanol extract of a single DG granule cell (C: representative nLC tracing). Black tracings: neurons; red tracings: artificial cerebrospinal fluid.

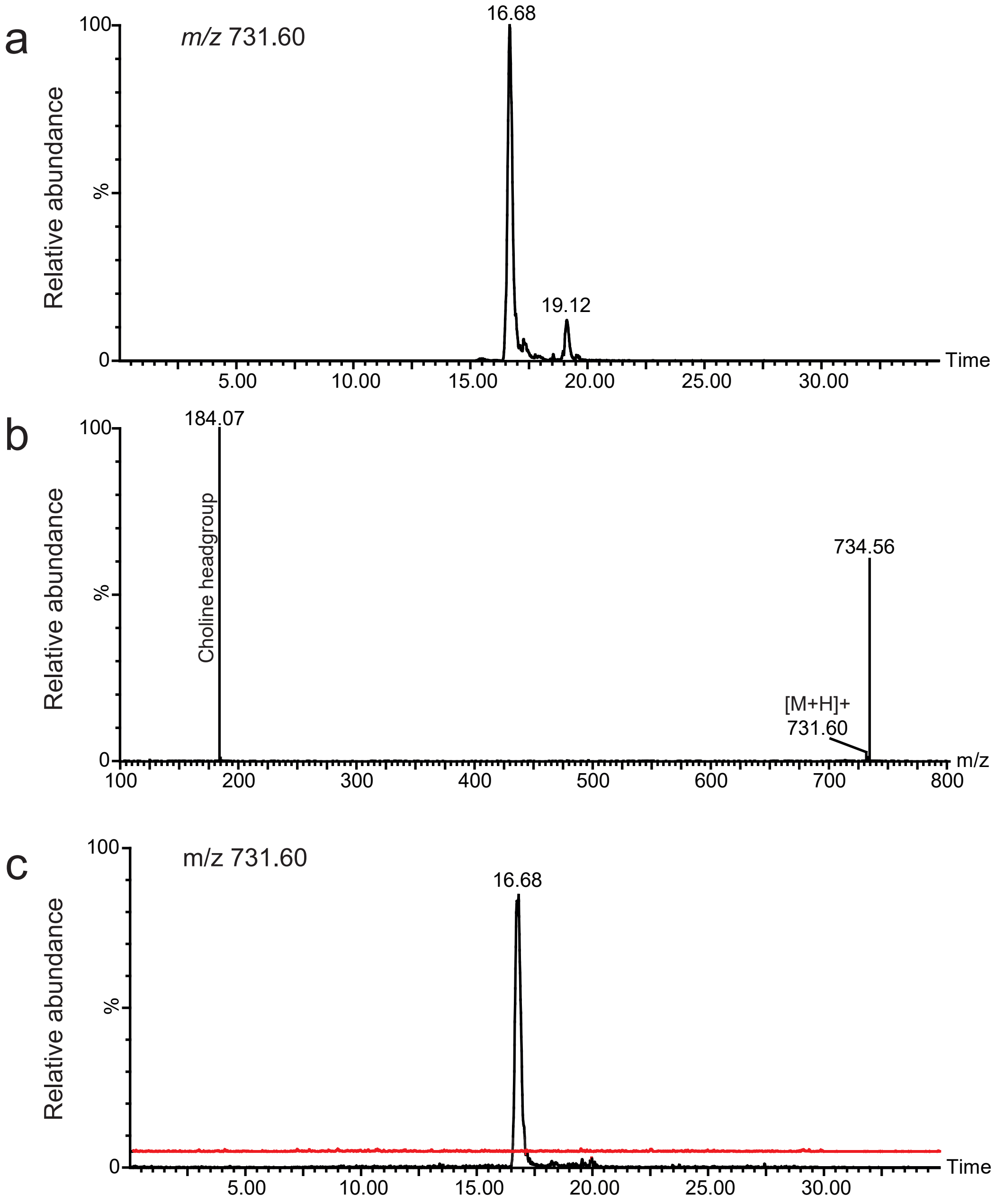

Supplementary Figure S35. Identification of SM (d18:1/18:0) (*m/z* = 731.60) in diluted extract (1:50,000) of whole hippocampal tissue (A: representative nLC tracing; B: mass spectrum) and isopropanol extract of a single DG granule cell (C: representative nLC tracing). Black tracings: neurons; red tracings: artificial cerebrospinal fluid.

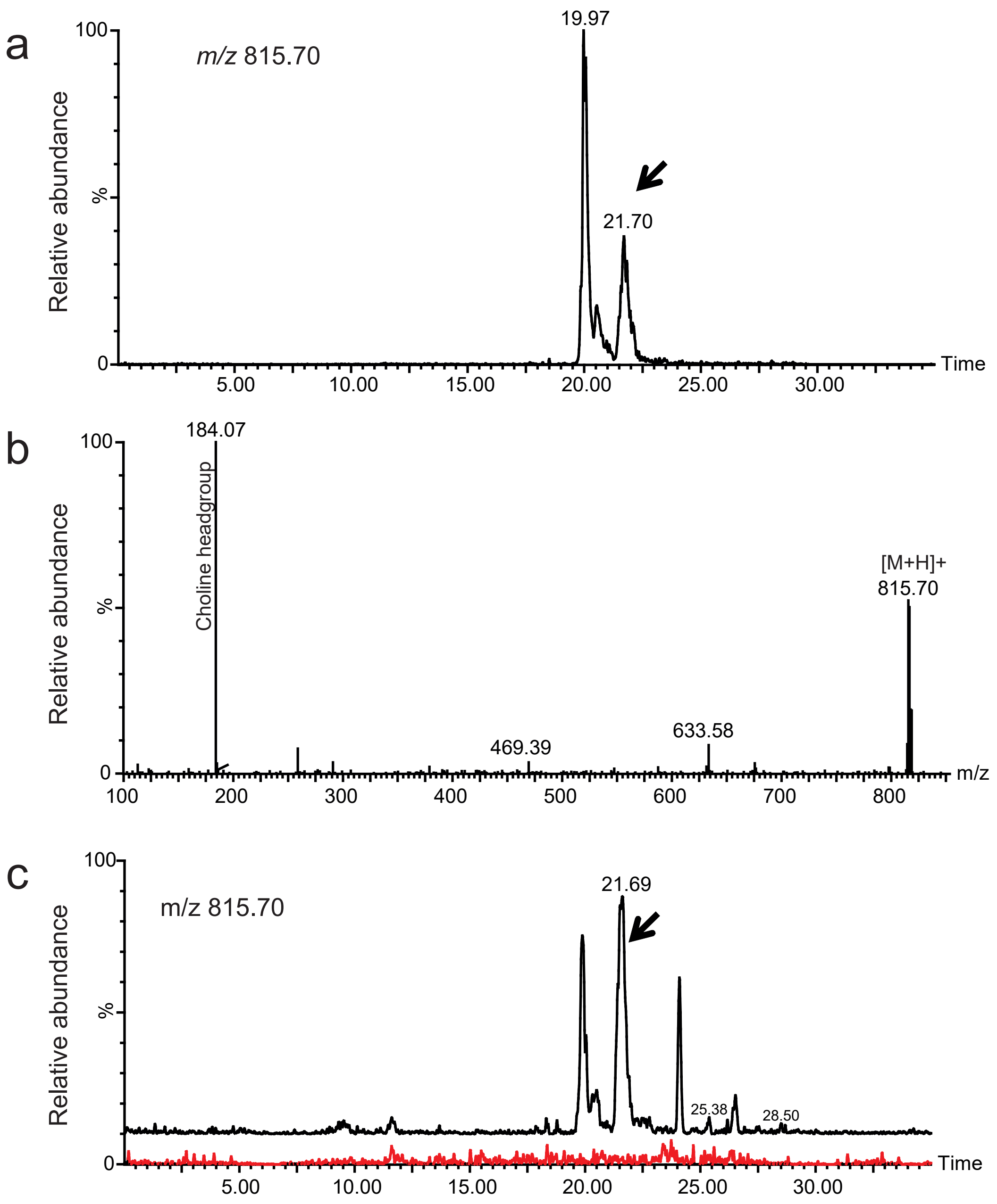

Supplementary Figure S36. Identification of SM (d18:1/24:0) (*m/z* = 815.70) in diluted extract (1:50,000) of whole hippocampal tissue (A: representative nLC tracing; B: mass spectrum) and isopropanol extract of a single DG granule cell (C: representative nLC tracing). Black tracings: neurons; red tracings: artificial cerebrospinal fluid.

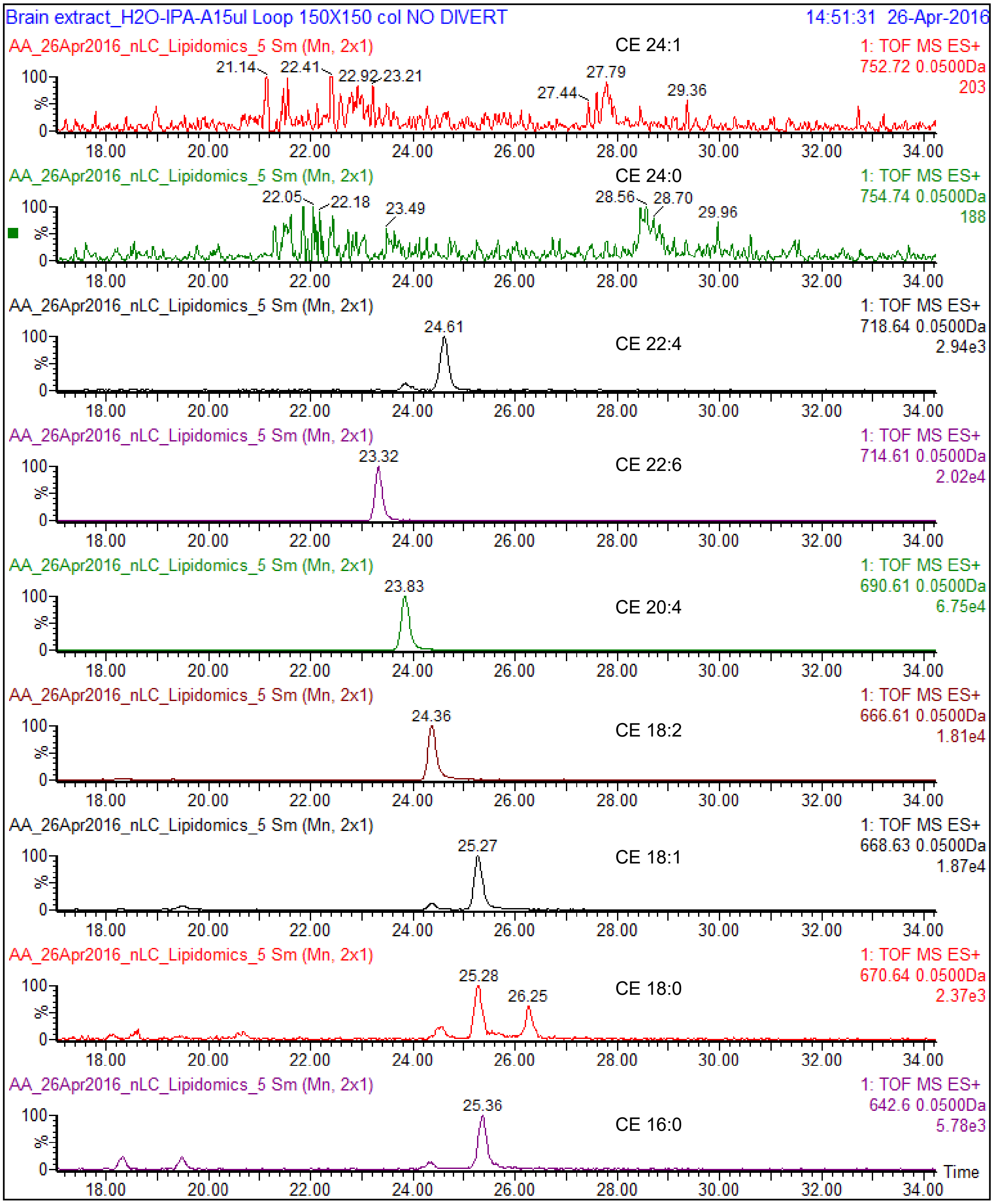

Supplementary Figure S37. Identification of cholesterol esters in diluted extract (1:50,000) of whole hippocampal tissue. Extracted ion currents (*m/z* = 369.35, cholesterol base peak) suggesting the presence of various cholesterol ester species in the extracts.

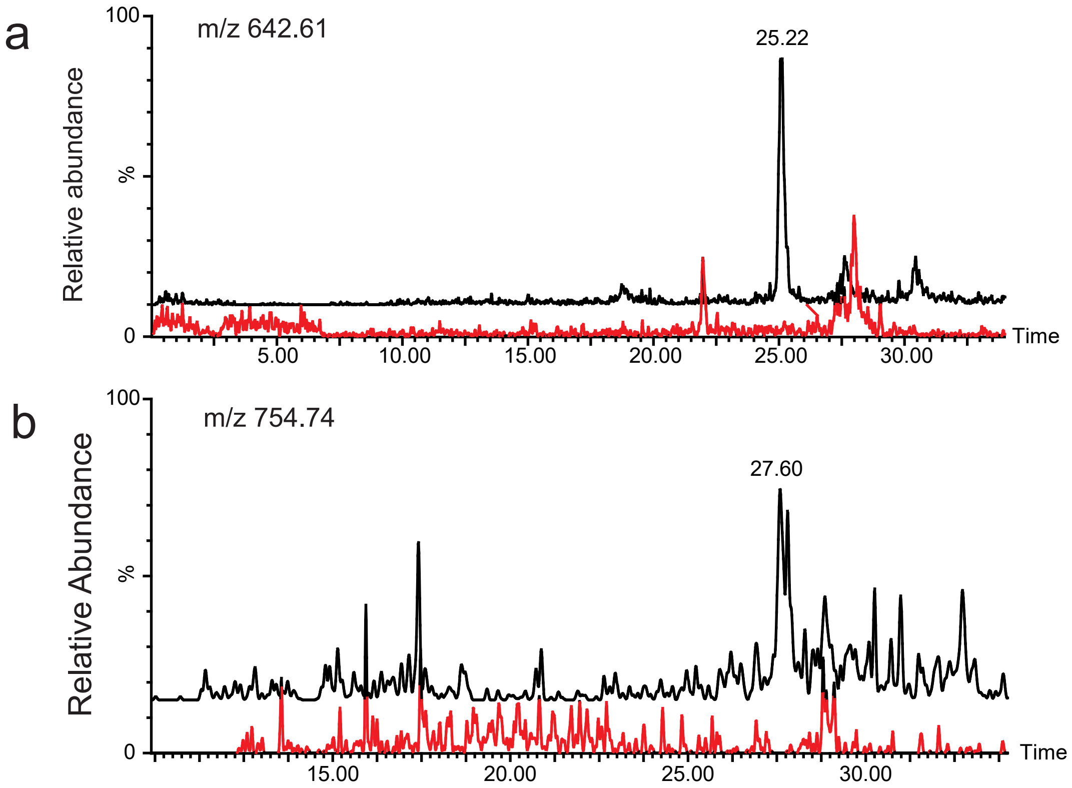

Supplementary Figure S38. Representative nLC tracings showing the identification of (A) CE 16:0 (*m/z* = 642.61) and (B) CE 24:0 (*m/z* = 754.74) in isopropanol extracts of a single DG granule cell. Black tracings: neurons; red tracings: artificial cerebrospinal fluid.

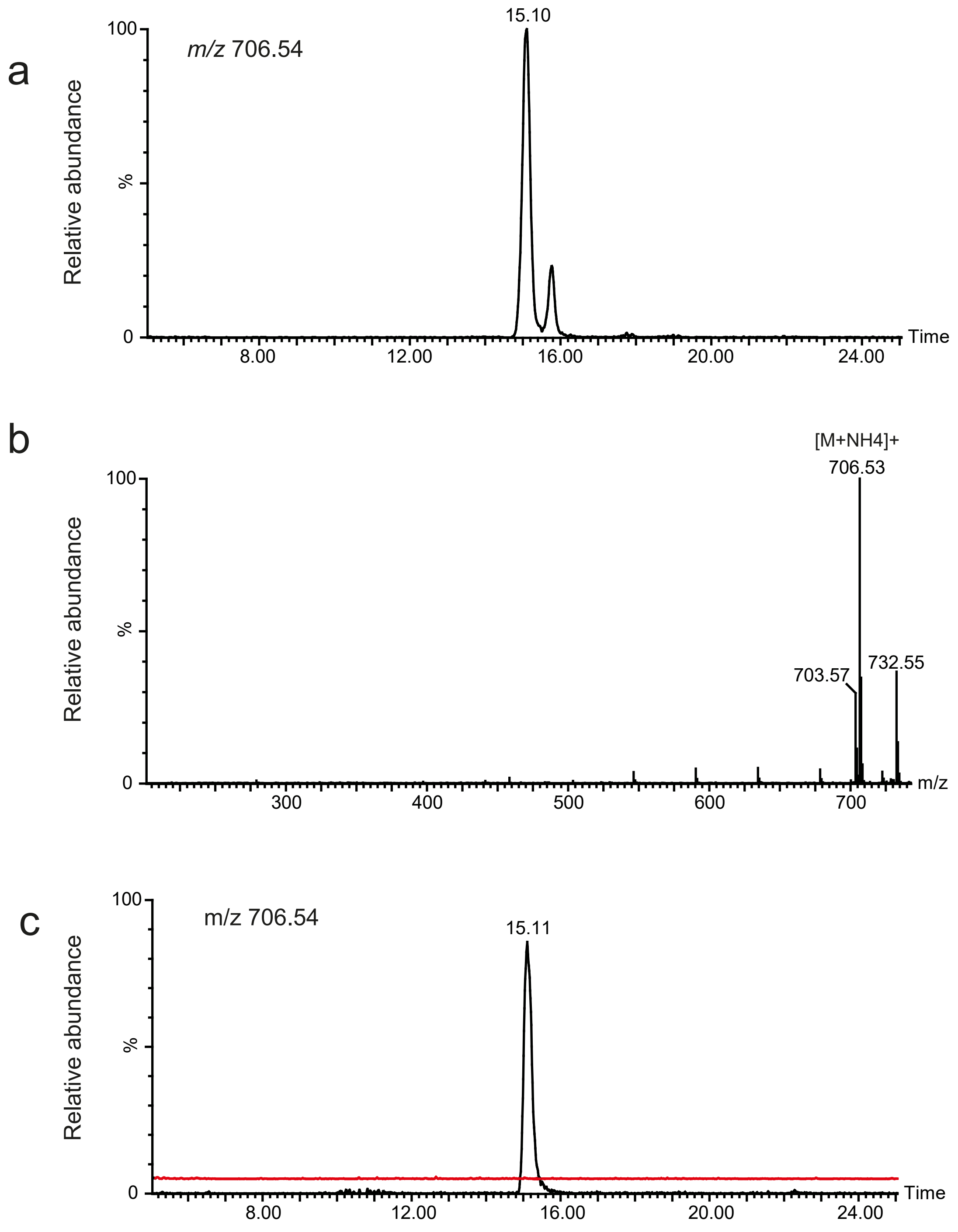

Supplementary Figure S39. Tentative identification of DAG 42:10 (*m/z* = 706.54) in diluted extract (1:50,000) of whole hippocampal tissue (A: representative nLC tracing; B: mass spectrum) and isopropanol extract of a single DG granule cell (C: representative nLC tracing). Because of possible coelution with isobaric lipids, identification must be considered tentative in absence of MS^2 data.

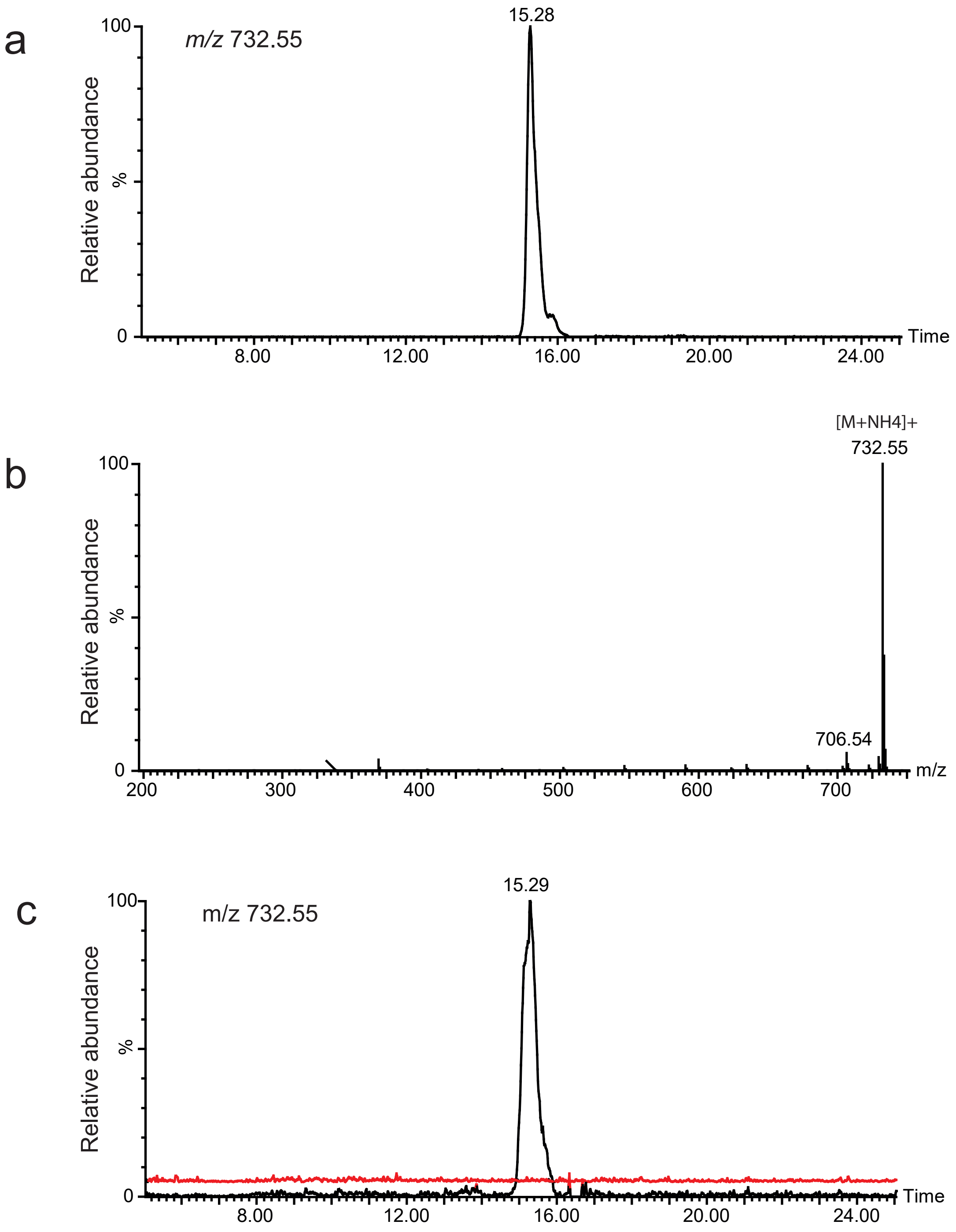

Supplementary Figure S40. Tentative identification of DAG 44:11 (*m/z* = 732.55) in diluted extract (1:50,000) of whole hippocampal tissue (A: representative nLC tracing; B: mass spectrum) and isopropanol extract of a single DG granule cell (C: representative nLC tracing). Because of possible coelution with isobaric PC species, identification must be considered tentative in absence of MS^2 data.

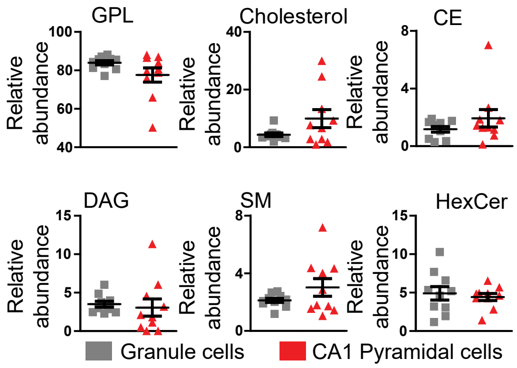

Supplementary Figure S41. Relative quantification of main lipid classes from non-stimulated individual DG granule cells (gray squares) and CA1 pyramidal cells (red triangles).

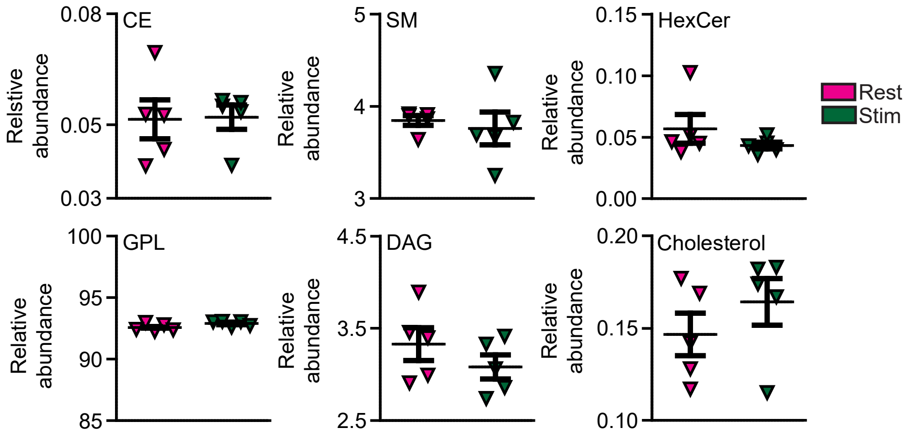

Supplementary Figure S42. Relative quantification of main lipid class-es from punches of non-stimulated (red triangles) and stimulated (green triangles) DG tissue.

Supplemental Table S1. Lipid species present in the Lipid Maps database, which were searched for in single-neuron extracts.				
**Lipid Species**	**[M+H]+**	**[M-H2O+H]+**	**[M+NH4]+**	**[M+Na]+**
**Sphingolipids**				
Cer (d18:1/16:0)	538.5194	520.5093	-	560.5013
Cer (d18:1/18:0)	566.5507	548.5406	-	588.5326
Cer (d18:1/20:0)	594.5820	576.5720	-	616.5639
Cer (d18:1/22:0)	622.6133	604.6033	-	644.5952
Cer (d18:1/24:0)	650.6466	632.6346	-	672.6285
Cer (d18:1/24:1)	648.6289	630.6189	-	670.6108
Cer (d18:0/16:0)	540.5350	522.5250	-	562.5169
Cer (d18:0/18:0)	568.5663	550.5563	-	590.5482
Cer (d18:0/20:0)	596.5976	578.5876	-	618.5795
Cer (d18:0/22:0)	624.6289	606.6189	-	646.6108
Cer (d18:0/24:0)	652.6602	634.6502	-	674.6421
Cer (d18:0/24:1)	650.6446	632.6346	-	672.6265
HexCer (d18:1/16:0)	700.5728	682.5622	-	722.5547
HexCer (d18:1/18:0)	728.6041	710.5935	-	750.5860
HexCer (d18:1/18:1)	726.6041	708.5935	-	748.5860
HexCer (d18:1/20:0)	756.6353	738.6248	-	778.6172
HexCer (d18:1/22:0)	784.6666	766.6561	-	806.6485
HexCer (d18:1/24:0)	812.6979	794.6874	-	834.6798
HexCer (d18:1/24:0-OH)	828.6923	810.6818	-	850.6742
HexCer (d18:1/24:1)	810.6823	792.6718	-	832.6642
SM (d18:1/16:0)	703.5780	-	-	725.5599
SM (d18:1/16:1)	701.5592	-	-	723.5411
SM (d18:1/18:0)	731.6061	-	-	753.5880
SM (d18:1/18:1)	729.5909	-	-	751.5728
SM (d18:1/20:0)	759.6374	-	-	781.6193
SM (d18:1/20:1)	757.6300	-	-	779.6119
SM (d18:1/22:0)	787.6687	-	-	809.6506
SM (d18:1/22:1)	785.6531	-	-	807.6350
SM (d18:1/24:0)	815.7000	-	-	837.6819
SM (d18:1/24:1)	813.6844	-	-	835.6663
SM (d18:1/26:0)	843.7313	-	-	865.7132
**Glycerolipids**				
MAG (16:0)	331.2843	-	348.3108	-
MAG (18:0)	359.3156	-	376.3421	-
MAG (18:1)	357.2999	-	374.3265	-
MAG (20:4)	379.2843	-	396.3108	-
MAG (22:6)	403.2843	-	420.3108	-
MAG (24:0)	443.4095	-	460.4360	-
MAG (24:1)	441.3939	-	458.4204	-
MAG (26:0)	471.4408	-	488.4673	-
DAG (32:0)	569.5139	-	586.5405	-
DAG (34:0)	597.5452	-	614.5718	-

DAG (34:1)	595.5296	-	612.5561	-
DAG (36:4)	617.5139	-	634.5405	-
DAG (38:6)	641.5139	-	658.5405	-
DAG (40:0)	681.6391	-	698.6657	-
DAG (40:1)	679.6235	-	696.6500	-
DAG (42:0)	709.6704	-	726.6970	-
DAG (36:0)	625.5765	-	642.6031	-
DAG (36:1)	623.5609	-	640.5874	-
DAG (38:4)	645.5452	-	662.5718	-
DAG (40:6)	669.5452	-	686.5718	-
DAG (42:0)	709.6704	-	726.6970	-
DAG (42:1)	707.6548	-	724.6813	-
DAG (44:0)	737.7017	-	754.7283	-
DAG (36:2)	621.5452	-	638.5718	-
DAG (38:5)	643.5296	-	660.5561	-
DAG (40:7)	667.5296	-	684.5561	-
DAG (42:2)	705.6391	-	722.6657	-
DAG (44:1)	735.6861	-	752.7126	-
DAG (36:4)	617.5139	-	634.5405	-
DAG (40:8)	665.5139	-	682.5405	-
DAG (42:8)	689.5139	-	706.5405	-
DAG (44:4)	729.6391	-	746.6657	-
DAG (44:5)	727.6235	-	744.6500	-
DAG (46:4)	757.6704	-	774.9700	-
DAG (38:6)	641.5139	-	658.5405	-
DAG (42:10)	689.5138	-	706.5405	-
DAG (44:12)	713.5139	-	730.5405	-
DAG (44:11)	715.5295	-	732.5561	-
DAG (46:6)	753.6391	-	770.6657	-
DAG (46:7)	751.6235	-	768.6500	-
DAG (48:6)	781.6704	-	798.6970	-
DAG (48:0)	793.7643	-	810.7909	-
DAG (48:1)	791.7487	-	808.7752	-
DAG (50:0)	821.7956	-	838.8222	-
**Cholesterol and Derivatives**				
Cholesterol	-	369.3514	404.3887	-
OH-Cholesterol	-	-	420.3836	-
CE (16:0)	-	-	642.6183	-
CE (16:1)	-	-	640.6027	-
CE (18:0)	-	-	670.6496	-
CE (18:1)	-	-	668.6340	-
CE (18:2)	-	-	666.6183	-
CE (18:3)	-	-	664.6027	-
CE (20:4)	-	-	690.6183	-
CE (22:6)	-	-	714.6183	-
CE (22:4)	-	-	718.6496	-
CE (24:0)	-	-	754.7446	-
**Phosphatidylinositol (PI)**				

PI (38:4)	887.5657	-	904.5922	-
PI (40:6)	911.5659	-	928.5918	-
PI (38:5)	885.5500	-	902.5760	-
PI (36:4)	859.5334	-	876.5611	-
PI (42:8)	907.5346	-	924.5593	-
PIP (34:2)	915.4994	-	932.5260	-
PIP (36:4)	939.5029	-	956.5256	-
PIP (36:1)	945.5464	-	962.5729	-
PIP (38:5)	965.5278	-	982.5447	-
PIP (38:4)	967.5322	-	984.5586	-
**Phosphatidylcholine (PC)**				
PC (32:0)	734.5694	-	-	756.5513
PC (34:0)	762.6007	-	-	784.5826
PC (36:0)	790.6320	-	-	812.6139
PC (38:0)	818.6633	-	-	840.6452
PC (40:0)	846.6946	-	-	868.6765
PC (42:0)	874.7259	-	-	896.7078
PC (34:1)	760.5851	-	-	782.5670
PC (34:2)	758.5694	-	-	780.5513
PC (34:3)	756.5538	-	-	778.5357
PC (34:4)	754.5381	-	-	776.5200
PC (38:1)	816.6477	-	-	838.6296
PC (38:2)	814.6320	-	-	836.6139
PC (38:3)	812.6164	-	-	834.5983
PC (38:4)	810.6007	-	-	832.5826
PC (38:5)	808.5851	-	-	830.5670
PC (38:6)	806.5694	-	-	828.5513
PC (36:1)	788.6164	-	-	810.5983
PC (36:2)	786.6007	-	-	808.5826
PC (36:3)	784.5890	-	-	806.5709
PC (36:4)	782.5694	-	-	804.5513
PC (40:1)	844.6790	-	-	866.6609
PC (40:2)	842.6570	-	-	864.6389
PC (40:3)	840.6477	-	-	862.6296
PC (40:4)	838.6320	-	-	860.6139
PC (40:5)	836.6164	-	-	858.5983
PC (40:6)	834.6007	-	-	856.5826
PC (42:1)	872.7102	-	-	894.6921
PC (52:12)	878.5694	-	-	900.5513
**Phosphatidylethanolamine (PE)**				
PE (32:0)	692.5225	-	-	714.5044
PE (34:0)	720.5538	-	-	742.5357
PE (36:0)	748.5851	-	-	770.5670
PE (38:0)	776.6164	-	-	798.5983
PE (40:0)	804.6477	-	-	826.6296
PE (42:0)	832.6790	-	-	854.6609
PE (34:1)	718.5382	-	-	740.5201
PE (34:2)	716.5225	-	-	738.5044

PE (34:3)	714.5069	-	-	736.4888
PE (34:4)	712.4912	-	-	734.4731
PE (38:1)	774.6008	-	-	796.5827
PE (38:2)	772.5851	-	-	794.5670
PE (38:3)	770.5695	-	-	792.5514
PE (38:4)	768.5538	-	-	790.5357
PE (38:5)	766.5382	-	-	788.5201
PE (38:6)	764.5225	-	-	786.5044
PE (36:1)	746.5695	-	-	768.5514
PE (36:2)	744.5538	-	-	766.5357
PE (36:3)	742.5421	-	-	764.5240
PE (36:4)	740.5225	-	-	762.5044
PE (40:1)	802.6321	-	-	824.6140
PE (40:2)	800.6101	-	-	822.5920
PE (40:3)	798.6008	-	-	820.5827
PE (40:4)	796.5851	-	-	818.5670
PE (40:5)	794.5695	-	-	816.5514
PE (40:6)	792.5538	-	-	814.5357
PE (40:6)	790.5381	-	-	812.5200
PE (42:1)	830.6633	-	-	852.6452
PE (42:2)	828.6477	-	-	850.6296
PE (52:12)	964.7729	-	-	986.7548
**Phosphatidylserine (PS)**				
PS (32:0)	736.5123	-	-	758.4942
PS (34:0)	764.5436	-	-	786.5255
PS (36:0)	792.5749	-	-	814.5568
PS (38:0)	820.6062	-	-	842.5881
PS (40:0)	848.6375	-	-	870.6194
PS (42:0)	876.6688	-	-	898.6507
PS (34:1)	762.5280	-	-	784.5099
PS (34:2)	760.5123	-	-	782.4942
PS (34:3)	758.4967	-	-	780.4786
PS (34:4)	756.4810	-	-	778.4629
PS (38:1)	818.5906	-	-	840.5725
PS (38:2)	816.5749	-	-	838.5568
PS (38:3)	814.5593	-	-	836.5412
PS (38:4)	812.5436	-	-	834.5255
PS (38:5)	810.5280	-	-	832.5099
PS (38:6)	808.5123	-	-	830.4942
PS (36:1)	790.5593	-	-	812.5412
PS (36:2)	788.5436	-	-	810.5255
PS (36:3)	786.5319	-	-	808.5138
PS (36:4)	784.5123	-	-	806.4942
PS (40:1)	846.6219	-	-	868.6038
PS (40:2)	844.5999	-	-	866.5818
PS (40:3)	842.5906	-	-	864.5725
PS (40:4)	840.5749	-	-	862.5568
PS (40:5)	838.5593	-	-	860.5412

PS (40:6)	836.5436	-	-	858.5255
PS (40:7)	834.5279	-	-	856.5098
PS (42:1)	874.6531	-	-	896.6350
PS (52:12)	1008.7627	-	-	1030.7446
**Fatty Acid Ethanolamides (FAE)**				
FAE (16:1)	298.2741	-	-	-
FAE (16:0)	300.2897	-	-	-
FAE (18:1)	326.3053	-	-	-
FAE (18:0)	328.321	-	-	-
FAE (20:5)	346.27	-	-	-
FAE (20:4)	348.2897	-	-	-
FAE (20:3)	350.3053	-	-	-
FAE (20:2)	352.31	-	-	-
FAE (20:1)	354.3367	-	-	-
FAE (20:0)	356.3523	-	-	-
FAE (22:6)	370.2744	-	-	-
FAE (22:5)	372.29	-	-	-
FAE (22:4)	376.321	-	-	-
FAE (22:1)	382.368	-	-	-
FAE (22:0)	384.3836	-	-	-
FAE (24:1)	410.3993	-	-	-
FAE (23:0)	398.3992	-	-	-
FAE (24:0)	412.4149	-	-	-
FAE (25:0)	426.4299	-	-	-
FAE (26:0)	440.4462	-	-	-
FAE (26:1)	438.4306	-	-	-
**N-Acyl Taurines (N-AcT)**				
N-AcT (16:1)	362.236	-	-	-
N-AcT (16:0)	364.2516	-	-	-
N-AcT (18:1)	390.2673	-	-	-
N-AcT (18:0)	392.2829	-	-	-
N-AcT (20:4)	412.2516	-	-	-
N-AcT (20:3)	414.2674	-	-	-
N-AcT (20:1)	418.2986	-	-	-
N-AcT (20:0)	420.3142	-	-	-
N-AcT (22:6)	436.2519	-	-	-
N-AcT (22:5)	438.2675	-	-	-
N-AcT (22:4)	440.2831	-	-	-
N-AcT (22:1)	446.3299	-	-	-
N-AcT (22:0)	448.3455	-	-	-
N-AcT (24:1)	474.3612	-	-	-
N-AcT (24:0)	476.3768	-	-	-
N-AcT (26:0)	504.4081	-	-	-
**Phosphatidic Acids (PA)**				
PA(34:1)	675.4959	-	692.5225	-
PA(36:2)	701.5116	-	718.5382	-
PA(34:2)	673.4803	-	690.5069	-

PA(32:0)	649.4803	-	666.5069	-
PA(34:0)	677.5116	-	694.5382	-
PA(36:0)	705.5429	-	722.5695	-
PA(36:1)	703.5272	-	720.5538	-
PA(36:2)	701.5115	-	718.5381	-
PA(36:3)	699.4958	-	716.5224	-
PA(36:4)	697.4801	-	714.5067	-
PA(38:0)	733.5742	-	750.6008	-
PA(38:1)	731.5585	-	748.5851	-
PA(38:4)	725.5114	-	742.538	-
PA(38:2)	729.5428	-	746.5694	-
PA(38:3)	727.5271	-	744.5537	-
Note: Ion adducts were selected based on data published in lipidmaps.org (35, 36).				
